# Secular trends in dietary energy, carbohydrate, protein and fat intake among Korean children and adolescents

**DOI:** 10.1017/S1368980024002180

**Published:** 2024-11-22

**Authors:** Da-In Sung, Yu-Jin Kwon, Seok-Jae Heo, Ji-Won Lee

**Affiliations:** 1 Department of Medicine, Yonsei University College of Medicine, Seoul, Republic of Korea; 2 Department of Family Medicine, Yongin Severance Hospital, Yonsei University College of Medicine, Gyeonggi, Republic of Korea; 3 Division of Biostatistics, Department of Biomedical Systems Informatics, Yonsei University College of Medicine, Seoul, Republic of Korea; 4 Department of Family Medicine, Severance Hospital, Yonsei University College of Medicine, Seoul, Republic of Korea; 5 Institute for Innovation in Digital Healthcare, Yonsei University, Seoul, Republic of Korea

**Keywords:** Children, Adolescents, Total calorie, Macronutrients, Korean national survey

## Abstract

**Objective:**

We aimed to analyse the evolving trends in macronutrient intake and dietary composition among Korean children and adolescents over a 10-year period.

**Design:**

We utilised cross-sectional data from the Korean National Health and Nutrition Examination Survey (KNHANES) spanning the years 2010–2020. Overall, the study included 11 861 participants aged 6–18 years who completed the 24-h dietary recall survey. Subsequently, we assessed trends in energy consumption and macronutrient intake across population subgroups, including age, sex and obesity status. Survey-weighted linear regression was employed to determine the *β* coefficient and *P*-value for trends in dietary nutrient consumption, treating the survey year as a continuous variable.

**Setting:**

KNHANES from 2010 to 2020.

**Participants:**

11 861 children and adolescents aged 6–18 years.

**Results:**

Total energy intake significantly decreased across the 10-year survey period, with a corresponding decline in the percentage of energy intake from carbohydrates. Conversely, the proportion of energy intake from fat increased during the same period. Subgroup analysis revealed changes in the composition of energy intake across age, sex and obesity status, with a consistent increase in total fat intake observed across all subgroups. Upon analysing data on dietary fibres, total sugars and fat subtypes intake, we found insufficient dietary fibre intake and increased intake of all fat subtypes.

**Conclusions:**

This study underscores the gradually changing dietary intake patterns among Korean children and adolescents. Our findings revealed that these transitions in dietary nutrient consumption may pose potential risks of diet-related diseases in the future.

Suboptimal diet is one of the leading risk factors for poor health outcomes, particularly contributing to a high risk of obesity, type 2 diabetes mellitus, cardiovascular diseases (CVD) and specific diet-related cancers^(1,2)^. Dietary risk factors, including excessive intake of saturated fats, refined carbohydrates and sweets, alongside inadequate intake of whole grains, fruits, vegetables and seafood, significantly contribute to the global burden of non-communicable diseases^(3,4)^. Thus, understanding the overall population trends in diet is crucial for recognising challenges and opportunities for improving nutritional status and reducing diet-related illnesses^(1,2)^.

The formative years of childhood and adolescence are pivotal for shaping dietary preferences and behaviours, exerting lasting effects on dietary patterns in adulthood and life-long susceptibility to chronic diseases^(3,5)^. During this developmental phase, dietary intake must meet energy demands and provide essential macronutrients to support vital processes in growth and development^(6)^. Recently, paediatric obesity has become a public concern owing to its association with various co-morbidities in childhood as well as obesity and related diseases in adulthood^(7)^. Globally, and particularly in Korea, there has been a surge in the prevalence of paediatric obesity^(8)^. According to the data from the National School Health Examination (NSHE) in Korea, the prevalence of obesity in children aged 6–18 years increased from 8·7 % in 2007 to 15·0 % in 2017^(9)^. Additionally, findings from a 30-year longitudinal cohort study in Korea (1986–2017) showed a significant rise in obesity rates, particularly among individuals aged 12 years and older^(10)^. This trend correlates with increased consumption of fast foods accompanied by sugary beverages and reduced intake of vegetables^(6,11)^.

Asian countries, including Korea, have witnessed substantial shifts in food consumption and dietary habits in the recent decades^(12)^. Traditional Asian dietary patterns characterised by low-fat and high-fibre foods have been supplanted by the consumption of foods rich in fat, sugar and salt, and these changes have had a profound impact on nutritional status and public health^(13,14)^ Specifically, these changes in dietary habits have resulted in nutritional imbalances among Korean children and adolescents, posing a significant concern during the growth period^(15)^. Therefore, it is necessary to identify recent changes in dietary consumption trends to develop strategies that can improve dietary patterns and alleviate disease burden. Despite this significance, studies examining secular trends in macronutrient consumption among children and adolescents at the population level in Korea are lacking.

Therefore, this study aimed to perform a comprehensive investigation of trends in dietary macronutrient intake among Korean children and adolescents over a 10-year period. Our major goal was to provide a more nuanced understanding of contemporary dietary trends by utilising data from the most recent decade. We also analysed the trends in macronutrient intake according to subgroups stratified by age, sex and obesity status. Furthermore, we examined the trends in the intake of dietary fibre, total sugars and fat subtypes to offer insights into the changes in dietary composition and highlight specific areas for nutrition interventions.

## Methods

### Study design and study population

Data for this study were sourced from the Korean National Health and Nutrition Examination Survey (KNHANES) performed from 2010 to 2020 to assess dietary nutrient intake. KNHANES is an annual nationwide cross-sectional survey conducted with nationally representative samples of Korea, consisting of non-institutionalised Korean citizens^(16)^. The KNHANES protocol received approval from the Institutional Review Board of the Korea Centres for Disease Control and Prevention, which has been detailed in a previous publication^(16)^.The study population included children and adolescents aged 6–18 years voluntarily participating in the survey. All participants provided informed consent, and the Institutional Review Board of Severance Hospital approved the study protocol (IRB number: 4-2022-0796). Finally, a total of 11 861 participants were included in the analysis.

### Assessment of dietary intake

In the KNHANES, dietary intake was assessed using a standardised 24-h dietary recall method. During the survey, participants reported all food and beverage consumed in the preceding 24 h through face-to-face interviews conducted by trained staff members at the participants’ households. Various measuring aids including food models and pictures were utilised to help participants recall detailed information on food and beverage consumption. Notably, the survey staff had undergone intensive training and participated in retraining sessions five to six times per year. In cases where the children could not respond independently, the primary respondents were permitted to request assistance from their main caregivers. Previous research on dietary assessment methodology has demonstrated the value of data collected through self-reporting methods in understanding dietary intake in children^(17)^. Subsequently, using the National Standard Food Composition Table from the Rural Development Administration, total energy, carbohydrates (g), dietary fibre (g), total sugars (g), proteins (g) and fatty acids (total fat, SFA, MUFA, PUFA, *n*-3 fatty acids (N3) and *n*-6 fatty acids (N6)) (g) were calculated^(18)^.

The percentage of energy intake from carbohydrates, total sugars and proteins were calculated using the following formula: carbohydrate (g), sugar (g) or protein (g) × 4 kcal/total energy intake (kcal) × 100. For fatty acids, the formula was as follows: fat (g) × 9 kcal/total energy intake (kcal) × 100. Notably, during the KNHANES from 2010 to 2015, information regarding the intake of total sugars was not collected. In addition, the intake of dietary fibre and fat subtypes (SFA, MUFA, PUFA, N3 and N6) was not evaluated during the 2010–2012 KNHANES. Detailed nutritional assessment information is available on the KNHANES website (https://knhanes.kdca.go.kr/knhanes).

### Covariates

Anthropometric measurements were collected during health examinations at mobile centres by trained medical personnel following standardised protocols. To ensure data accuracy, a quality control project was implemented through on-site inspections. The detailed measurement protocol has been previously documented^(19)^.

Obesity was defined using age- and sex-specific BMI reference values from the 2017 Korean National Growth Charts^(20)^. It was classified into two categories: obesity (based on weight) and obesity (based on BMI), which were defined as weight and BMI at or above the 95th percentile for age and sex, respectively^(20)^. Abdominal obesity was defined as a waist circumference (WC) at or above the 90th percentile of sex-specific WC for age^(21)^. Socio-economic status was investigated via a self-reported questionnaire and included household income, education level and residence. Household income was divided into quartiles, graded from the lowest to the highest. The level of education was classified as less than elementary school, middle school and high school. The residence area was determined based on participants’ living location, categorised as either ‘dong’ for urban areas or a ‘eup’/‘myeon’ for rural areas according to the administrative divisions of Korea.

### Study outcomes

The main outcome was energy intake from macronutrients, specifically carbohydrates, protein and fat. Subgroup analysis were conducted across various population subgroups, including age categories (6–8, 9–11, 12–14 and 15–18 years), sex, obesity (based on weight), obesity (based on BMI) and abdominal obesity. Additionally, trends in the intake of total sugars and fat subtypes were examined.

### Statistical analysis

Statistical analysis was conducted for data from 1 January 2010 to 31 December 2020. The sampling plan in the KNHANES follows a multi-stage clustered probability design. To address the complex sampling design, all survey statistics were evaluated utilising sample weights allocated to the sample participants. By incorporating sample weights into the analysis, parameters for the study population could be estimated accurately. Continuous variables are presented as mean ± se, whereas categorical variables are expressed as counts (percentages). Differences in variables between years were compared using one-way ANOVA and Pearson’s *χ* ^2^ test. We employed survey-weighted linear regression to calculate the *β* coefficient and *P*-value for linearly increasing or decreasing trends in dietary nutrient consumption, treating the survey year as a continuous variable. Additionally, the statistical significance of overall and trend differences in dietary intake was assessed using the Rao–Scott *χ* ^2^ test for subgroups including age, sex, obesity (based on weight), obesity (based on BMI) and abdominal obesity.

All statistical tests were conducted using R software (version 4.1.2; www.r-project.org; R Foundation for Statistical Computing). The statistical significance level was set at *P* < 0·05. We used the survey R package to perform the analysis with a complex sampling design.

## Results

### Participant characteristics

A total of 11 861 children and adolescents aged 6–18 years (6189 boys (52·2 %) and 5672 girls (47·8 %)) were included in this study (Table 1). The percentage of adolescents aged 9–11 years declined from 28 % in 2010 to 25 % in 2020. Additionally, the proportion of participants with the lowest household income decreased from 14·1 % in 2010 to 6·1 % in 2020, whereas those with the highest household income increased from 25·5 to 31·6 %. The number of participants living in rural areas decreased from 19 to 9·3 % over the 10-year period. Regarding anthropometric measurements, the mean BMI and WC increased from 19·6 kg/m^2^ to 20·1 kg/m^2^ and from 65·7 cm to 67·9 cm, respectively, over the 10-year span. The proportion of participants with general obesity (a combination of two types of obesity: weight and BMI) increased from 5·6 to 6·5 % over 10 years, while the proportion of participants with abdominal obesity rose from 5·5 to 6·5 %.

**Table 1.** Demographic and clinical characteristics of the study population (*n* 11 816) from 2010 to 2020†

Characteristic	2010		2011		2012		2013		2014		2015		2016		2017		2018		2019		2020		*P*-value*
	*n* or mean	% or se	*n* or mean	% or se	*n* or mean	% or se	*n* or mean	% or se	*n* or mean	% or se	*n* or mean	% or se	*n* or mean	% or se	*n* or mean	% or se	*n* or mean	% or se	*n* or mean	% or se	*n* or mean	% or se	
Total no. (*n* 11 816)	1460		1269		1160		1278		992		964		1073		1005		929		1010		721		
Sex, *n* (%)																							0·914
Male	776	53 %	663	52 %	618	53 %	666	52 %	514	52 %	509	53 %	553	52 %	505	50 %	477	51 %	518	51 %	390	54 %	
Female	684	47 %	606	48 %	542	47 %	612	48 %	478	48 %	455	47 %	520	48 %	500	50 %	452	49 %	492	49 %	331	46 %	
Age, *n* (%) (years)																							<0·001
6–8	368	25 %	344	27 %	255	22 %	297	23 %	258	26 %	224	23 %	315	29 %	275	27 %	247	27 %	281	28 %	195	27 %	
9–11	408	28 %	302	24 %	301	26 %	338	26 %	247	25 %	221	23 %	249	23 %	271	27 %	260	28 %	272	27 %	183	25 %	
12–14	374	26 %	311	25 %	318	27 %	298	23 %	226	23 %	247	26 %	240	22 %	209	21 %	203	22 %	212	21 %	164	23 %	
15–18	310	21 %	312	25 %	286	25 %	345	27 %	261	26 %	272	28 %	269	25 %	250	25 %	219	24 %	245	24 %	179	25 %	
Household income (quartile), *n* (%)																							<0·001
Quartile 1	147	10 %	140	11 %	99	8·6 %	133	10 %	93	9·5 %	120	12 %	98	9·2 %	86	8·6 %	77	8·3 %	93	9·2 %	48	6·7 %	
Quartile 2	394	27 %	353	28 %	282	25 %	375	30 %	240	24 %	224	23 %	273	26 %	253	25 %	236	25 %	299	30 %	198	28 %	
Quartile 3	442	31 %	432	34 %	399	35 %	386	30 %	392	40 %	320	33 %	336	31 %	337	34 %	340	37 %	317	31 %	256	36 %	
Quartile 4	457	32 %	329	26 %	367	32 %	374	29 %	259	26 %	296	31 %	363	34 %	329	33 %	275	30 %	299	30 %	218	30 %	
Education, *n* (%)																							0·225
< Elementary school	1148	83 %	935	80 %	848	80 %	924	79 %	714	79 %	695	78 %	800	79 %	748	80 %	697	81 %	758	81 %	544	80 %	
Middle school	197	14 %	209	18 %	185	17 %	218	19 %	171	19 %	170	19 %	180	18 %	167	18 %	138	16 %	156	17 %	111	16 %	
High school	31	2·3 %	31	2·6 %	25	2·4 %	32	2·7 %	23	2·5 %	24	2·7 %	27	2·7 %	23	2·5 %	26	3·0 %	25	2·7 %	29	4·2 %	
Residence, *n* (%)																							<0·001
Urban	1243	85 %	1099	87 %	998	86 %	1032	81 %	824	83 %	825	86 %	896	84 %	891	89 %	787	85 %	833	82 %	621	86 %	
Rural	217	15 %	170	13 %	162	14 %	246	19 %	168	17 %	139	14 %	177	16 %	114	11 %	142	15 %	177	18 %	100	14 %	
Total no. excluding missing values of WC (*n* 11 047)	1365		1177		1070		1189		923		894		1008		938		861		937		685		
WC, mean (se)	64·1	10·7	64·3	10·9	64·5	10·2	64·3	10·8	64·5	11·1	67·4·	11·9	65·0·	11·6	64·4·	11·3	65·4·	11·3	65·7·	12·2	67·6·	12·9	<0·001
Abdominal obesity	35	2·4 %	37	2·9 %	19	1·6 %	27	2·1 %	28	2·8 %	45	4·7 %	43	4·0 %	40	4·0 %	33	3·6 %	50	5·0 %	46	6·4 %	>0·999
Total no. excluding missing values of weight (*n* 11 065)	1371		1178		1070		1191		923		894		1010		938		862		943		685		
Weight, mean (se)	43·7	16·2	43·9	16·7	45·0	15·8	44·7	16·6	44·1	17·0	46·7	17·9	43·7	17·4	43·6	16·9	44·6	17·7	44·4	18·1	46·6	19·0	<0·001
Obesity	57	4·2 %	56	4·8 %	39	3·6 %	40	3·4 %	41	4·4 %	51	5·7 %	50	5·0 %	58	6·2 %	58	6·7 %	62	6·6 %	63	9·2 %	>0·999
Total no. excluding missing values of BMI (*n* 11 063)	1371		1178		1070		1191		923		894		1010		938		861		942		685		
BMI, mean (se)	19·2	3·6	19·2	3·8	19·2	3·5	19·3	3·7	19·3	3·9	19·8	4·2	19·1	4·0	19·2	3·9	19·4	4·1	19·5	4·3	20·1	4·5	<0·001
Obesity	64	4·7 %	56	4·8 %	34	3·2 %	48	4·0 %	37	4·0 %	52	5·8 %	51	5·0 %	48	5·1 %	55	6·4 %	61	6·5 %	63	9·2 %	>0·999

WC, waist circumference.

* *P*-values were calculated using one-way ANOVA or Pearson’s *χ* ^2^ test.

† General obesity was classified into two categories: obesity (weight) and obesity (BMI), defined as weight and BMI at or above the 95th percentile for age and sex, respectively. Abdominal obesity was defined as a WC at or above the 90^th^ percentile of sex-specific WC for age.

### Trends in carbohydrate, protein and fat intake

A total of 11 861 participants who completed the 24-h dietary recall survey were included in the nutrient analysis. As shown in Fig. 1, from 2010 to 2020, the total energy intake per d decreased from 2065·3 kcal/d in 2010 to 1842·7 kcal/d in 2020 (*β* coefficient = –17·25, *P* < 0·001). Similarly, the percentage of energy intake from carbohydrates decreased from 61·9 to 58·8 % (*β* coefficient = –0·38, *P* < 0·001). Conversely, the percentage of energy intake from protein and fat increased from 14·1 to 15·0 % (*β* coefficient = 0·08, *P* < 0·001) and 24·0 % to 26·2 % (*β* coefficient = 0·30, *P* < 0·001), respectively (see online supplementary material, Supplemental Table S1).

**Figure 1.**
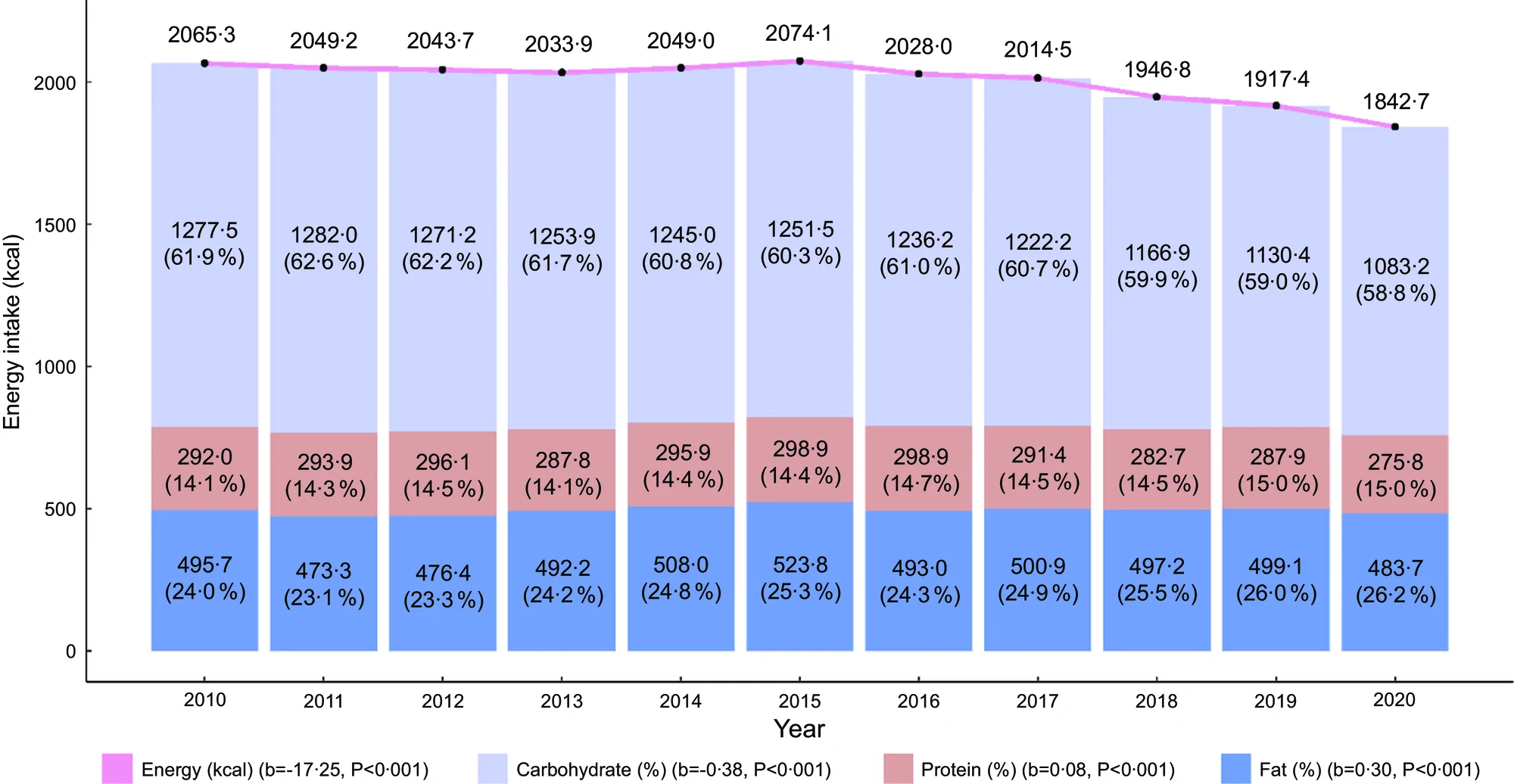
Total energy intake per d and trends in carbohydrate, protein and fat intake from 2010 to 2020 (*n* 11 816)*. *Survey-weighted linear regression was used to obtain *β* coefficients and *P*-values.

### Subgroup analysis

Trends in total energy intake and intake of carbohydrates (%), protein (%) and fat (%) across the population subgroups were examined (Figs. 2 and 3). Figure 2 shows the trends in energy intake and the percentage of macronutrient intake by age group and sex. Generally, energy intake increased with age, excluding the years 2014, 2019 and 2020, during which the energy intake of the 12–14-year age group exceeded that of the 15–18-year age group. Significant decreasing trends were observed in total energy intake in all age groups, excluding participants aged 6–8 years (9–11-year age group; *P* = 0·012, 12–14-year age group; *P* = 0·016, and 15–18-year age group; *P* < 0·001). These decreasing trends were consistent with the carbohydrate intake (%) in all age groups (all *P* < 0·001). Conversely, the intake of protein (%) and fat (%) increased in all age groups (all *P* < 0·01). Notably, the increase in fat intake (%) in the 15–18-year age group was 19 % (*P* = 0·004), whereas it was approximately half of that in other age groups (all *P* < 0·001, *P* for interaction = 0·031). Compared with girls, boys consumed more total energy and protein (%) (both *P* < 0·001) and less carbohydrates (%) (*P* = 0·008). However, no significant difference was observed in fat intake (%) between the sexes (*P* = 0·354). In both sexes, total energy intake and the proportion of carbohydrate intake (%) declined, whereas the intake of protein (%) and fat (%) increased over the 10-year period (all *P* < 0·001). Changes in total calorie, carbohydrate (%), protein (%) and fat (%) intake were not significantly different between the sexes (*P* = 0·590, *P* = 0·666, *P* = 0·235, *P* = 0·982, respectively) (see online supplementary material, Supplemental Table S2).

**Figure 2.**
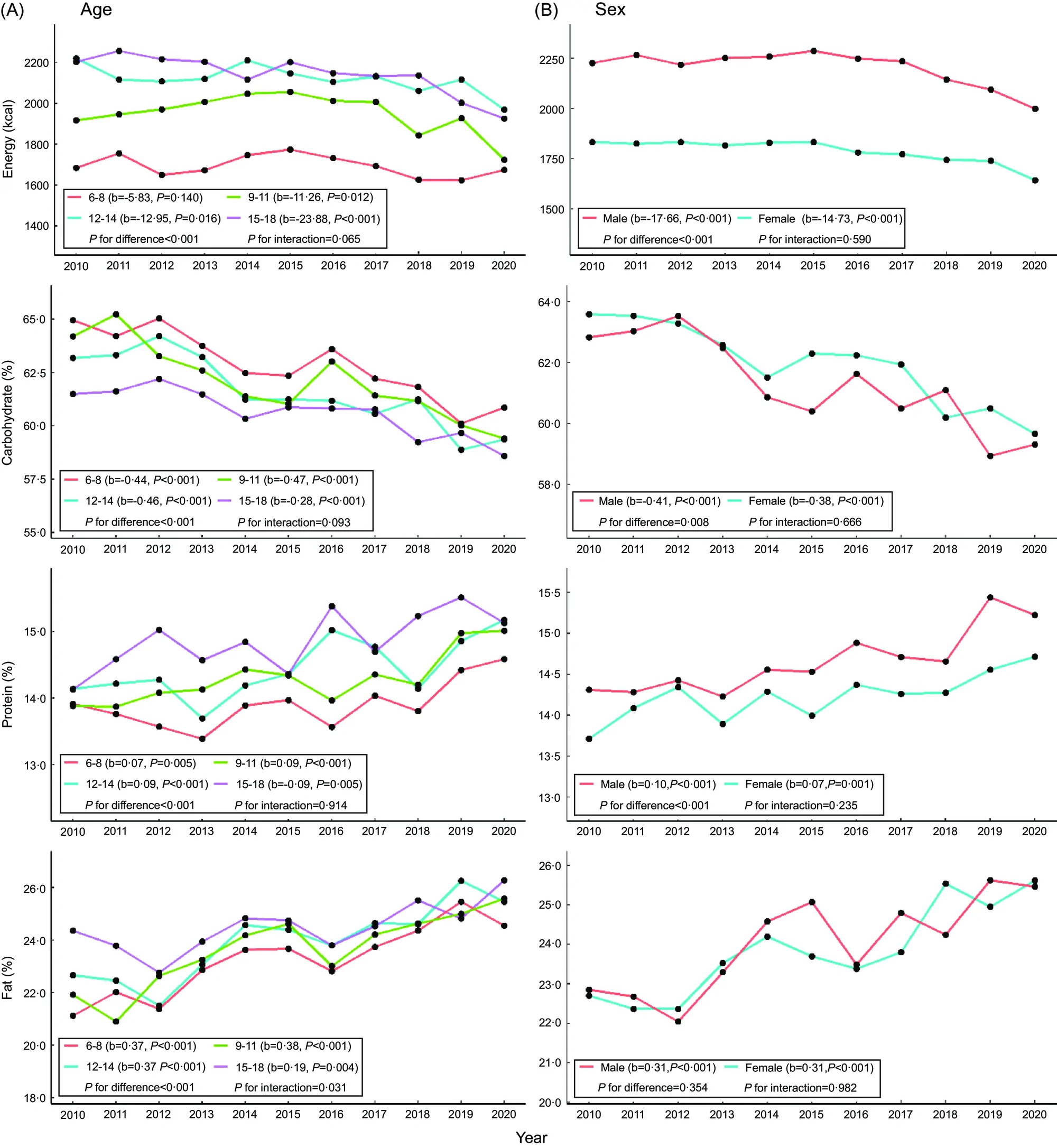
Trends in total energy and macronutrient intake according to (A) age and (B) sex (*n* 11 816)*. *Survey-weighted linear regression was used to obtain *β* coefficients and *P*-values. Rao–Scott *χ*^2^ test was used to obtain *P*-values for difference and interaction between groups.

**Figure 3.**
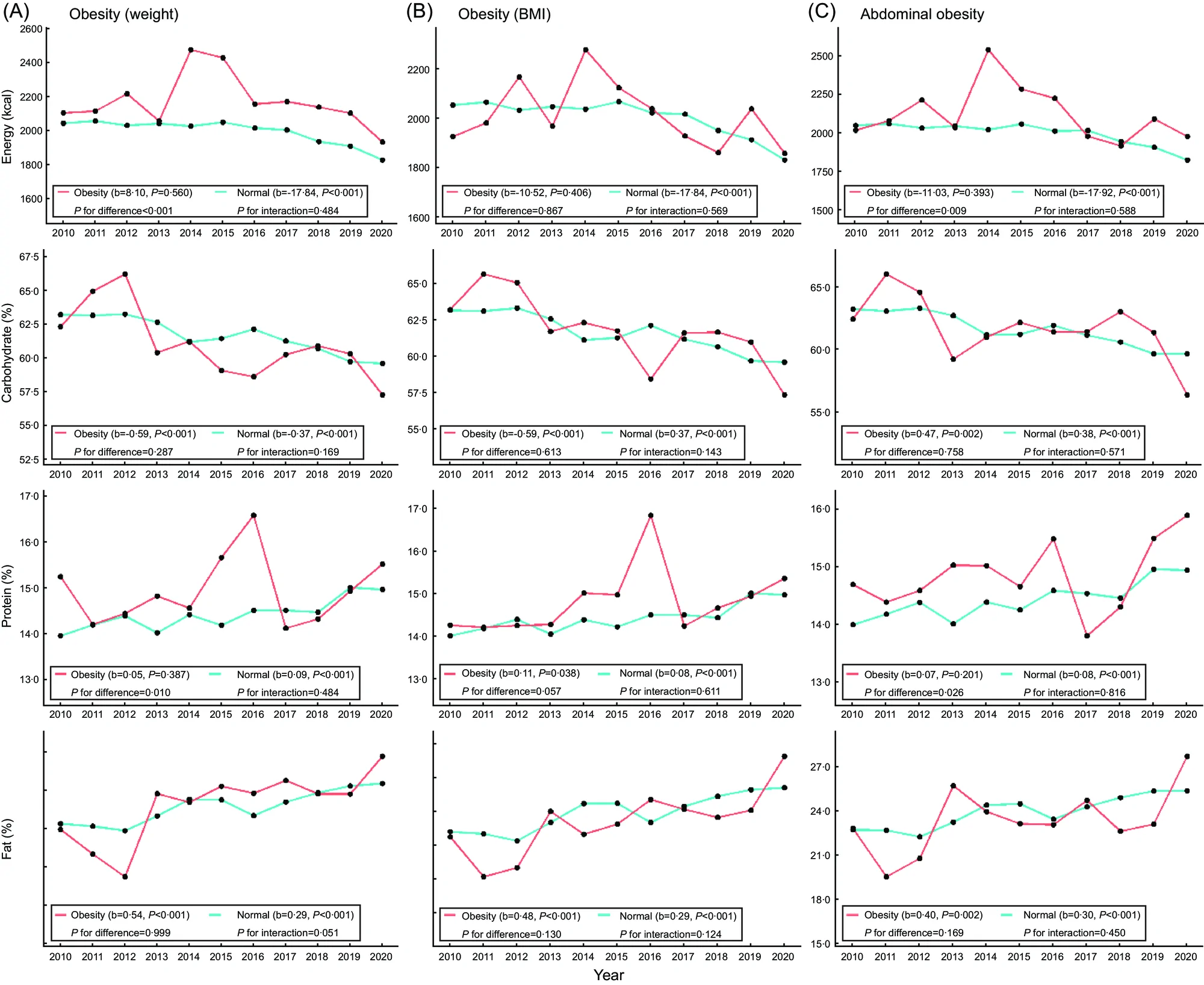
Trends in total energy and macronutrient intake according to the presence of (A, B) general or (C) abdominal obesity (n (A) = 11 065, n (B) = 11 063 and n (C) = 11 047)*†. *Survey-weighted linear regression was used to obtain *β* coefficients and *P*-values. Rao–Scott *χ* ^2^ test was used to obtain *P*-values for difference and interaction between groups. †General obesity was classified into two categories: obesity (weight) and obesity (BMI), which were defined as weight and BMI at or above the 95th percentile for age and sex, respectively. Abdominal obesity was defined as a waist circumference (WC) at or above the 90th percentile of sex-specific WC for age.

Figure 3 shows the trends in the total energy intake and dietary macronutrient composition according to the presence of general obesity (defined as weight and BMI for age) and abdominal obesity. Participants with obesity (weight) consumed higher total calories and protein (%) (*P* < 0·001 and *P* = 0·010, respectively), with no interaction noted. However, this trend of significantly higher consumption of total calories and protein (%) was not observed in participants with obesity (based on BMI). Nonetheless, similar trends were observed in participants with obesity (based on weight) and those with abdominal obesity (*P* = 0·009 and *P* = 0·026, respectively). Regardless of the presence of general or abdominal obesity, there was a significant increasing trend in fat intake (%) across all participants (abdominal obesity, *P* = 0·002; others, *P* < 0·001).

Participants with missing data on the intake of total sugars (2010–2015) and dietary fibre and fat subtypes (2010–2012) were excluded from the analysis. Figure 4 presents the overall trends in the percentage of energy intake from the total sugars between 2016 and 2020 and fat subtypes (SFA, MUFA, PUFA, N3 and N6) between 2013 and 2020. Energy intake from total sugars significantly decreased from 14·5 % in 2016 to 13·5 % in 2020 (*β* coefficient = –0·23, *P* = 0·030). However, there were significant increasing trends in the consumption of all fat subtypes over the 5 years (all *P* < 0·001). Trends in intake of total sugars (%) and fat (%) are delineated by age group and sex (Figs. 5 and 6). When analysed by age group, similar increasing trends in fat intake (%) were observed (all *P* < 0·05); however, the decreasing trend in total sugars intake (%) was only found in participants aged 6–8 years (*β* coefficient = –0·42, *P* = 0·010). Moreover, an increasing trend in dietary fibre intake was only observed in participants aged 12–14 years (*β* coefficient = 0·30, *P* = 0·019) (see online supplementary material, Supplemental Fig. S1). There were no significant interactions between the intake of total sugars (%) and fat (%) according to age group. In terms of sex, there were increasing trends in fat intake (%) in both boys and girls (all *P* < 0·001). However, the result was significant, excluding the percentage of N3 intake in girls (*β* coefficient = 0·01, *P* = 0·056). Changes in the consumption of N3 (%) were greater in boys than in girls (*P* = 0·046). There were no significant interactions between the intake of total sugars (%) and other fats (%) and sex. Furthermore, dietary fibre intake between 2013 and 2020 was 18·1–21·6 g/d in boys and 15·6–18·4 g/d in girls (see online supplementary material, Supplemental Table S3).

**Figure 4.**
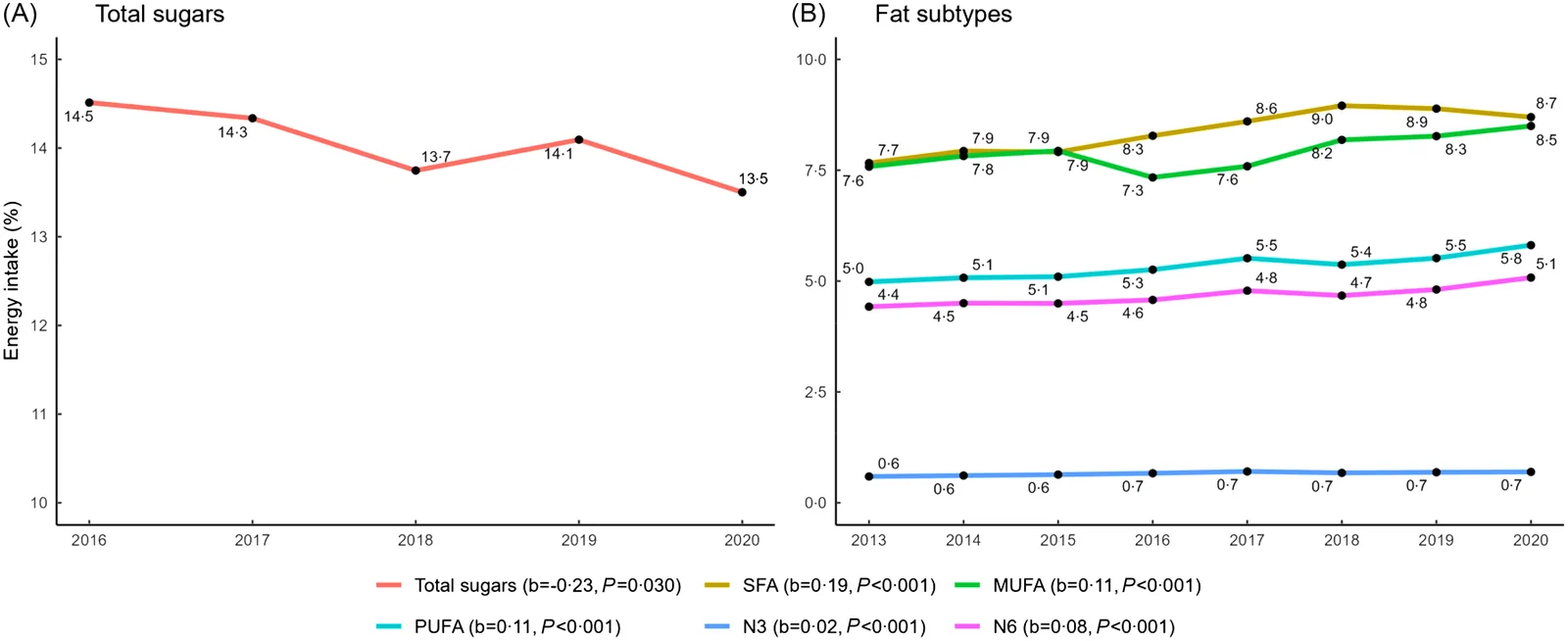
Overall trends in energy intake from total sugars and fat subtypes from 2016 to 2020 (n (A) = 4738 and n (B) = 7972)*. *Survey-weighted linear regression was used to obtain *β* coefficients and *P*-values. N3, *n*-3 fatty acids; N6, *n*-6 fatty acids.

**Figure 5.**
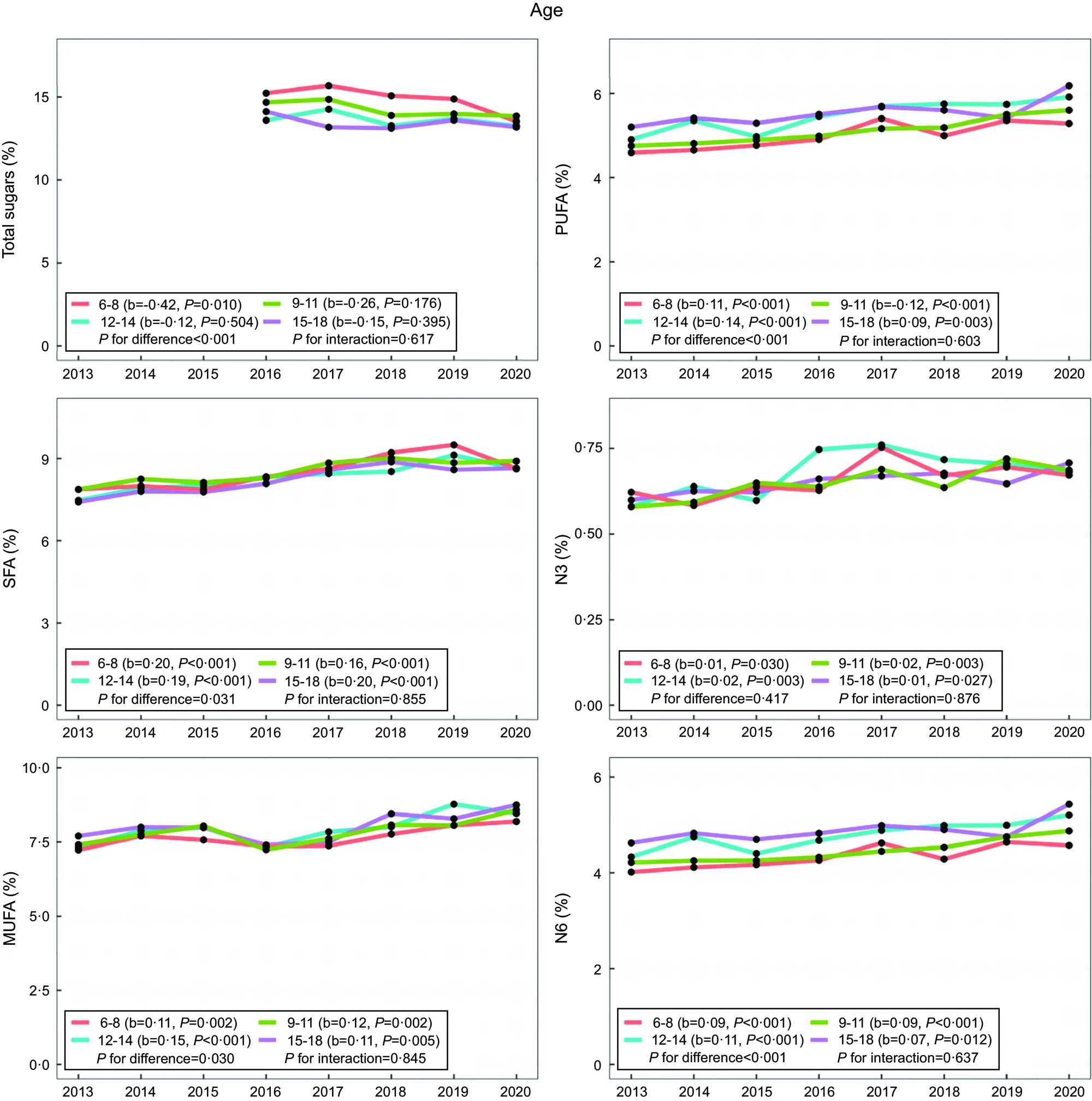
Trends in energy intake from total sugars and fat subtypes according to age group (*n* 4738 for total sugars and 7972 for fat subtypes)*. *Survey-weighted linear regression was used to obtain *β* coefficients and *P*-values. Rao–Scott *χ* ^2^ test was used to obtain *P*-values for difference and interaction between groups. N3, *n*-3 fatty acids; N6, *n*-6 fatty acids.

**Figure 6.**
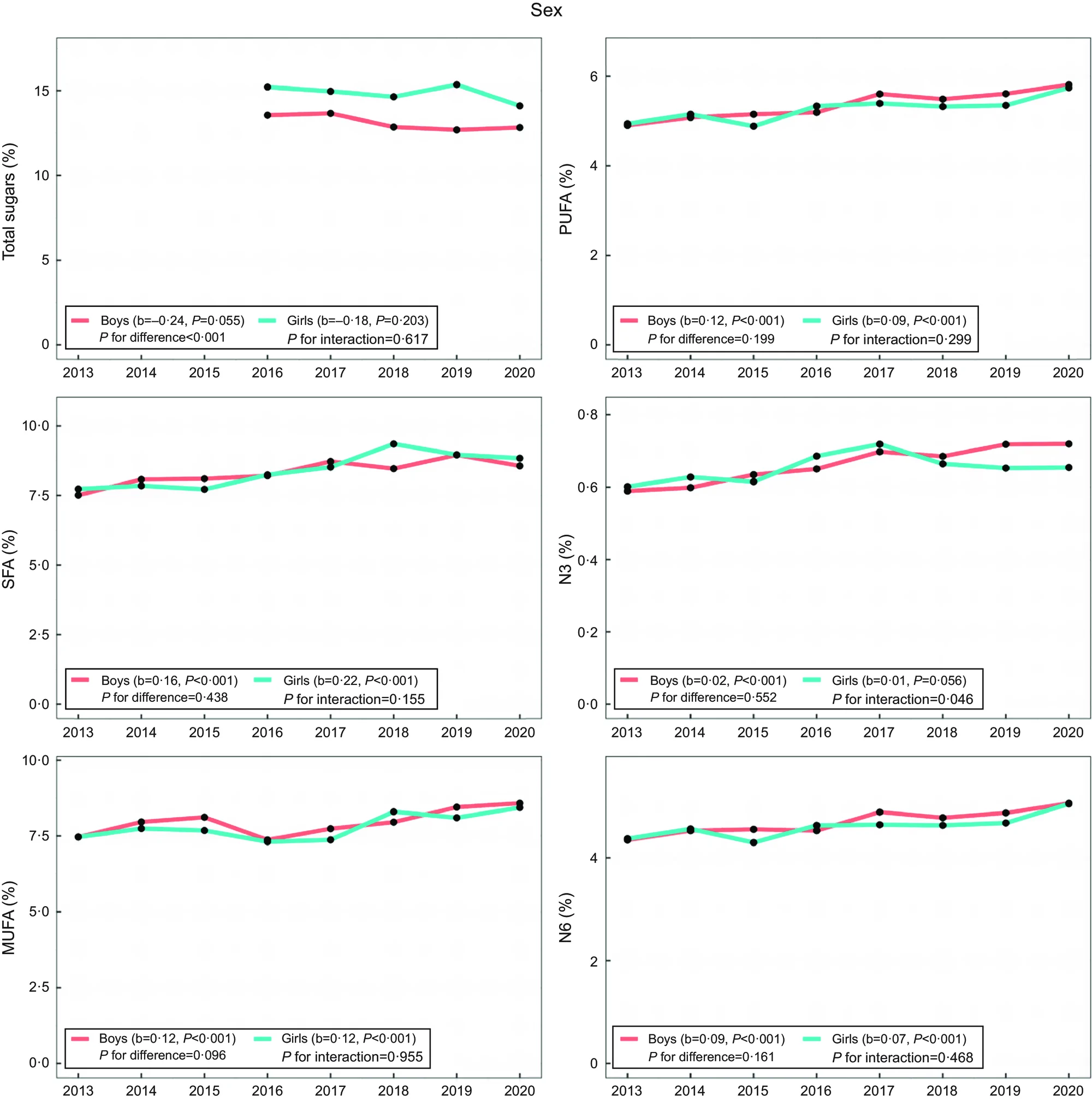
Trends in energy intake from total sugars and fat subtypes according to sex (*n* 4738 for total sugars and 7972 for fat subtypes)*. *Survey-weighted linear regression was used to obtain *β* coefficients and *P*-values. Rao–Scott *χ* ^2^ test was used to obtain *P*-values for difference and interaction between groups. N3, *n*-3 fatty acids; N6, *n*-6 fatty acids.

## Discussion

Utilising nationally representative data from 2010 to 2020, this study represents the first attempt to describe the secular trends in dietary energy and macronutrient intake among Korean children and adolescents, examining differences across subgroups defined by age, sex and obesity status. We observed decreasing trends in total energy intake alongside a greater proportion of fat in dietary nutrient intake. Additionally, the increase in total fat intake corresponded to increasing trends in the consumption of five fat subtypes (SFA, MUFA, PUFA, N3 and N6). These findings indicate a dietary nutrient transition among Korean children and adolescents over the 10-year period, highlighting the need for continuous monitoring and public health consideration.

This study revealed a decrease in daily total energy intake between 2010 and 2020. First, the observed decline in total energy intake among Korean children and adolescents may be associated with their dietary environment, as childhood dietary habits are predominantly influenced by parental dietary lifestyle factors^(22)^. Moreover, the significant decrease in total energy intake aligns with a recent analysis of dietary macronutrient intake trends in Korean adults^(19)^, suggesting a potential correlation among family members’ consumption patterns^(23)^. Second, this finding may be related to the pattern of skipping breakfast, which has recently appeared more prevalent among Korean children and adolescents^(6)^. Despite the fundamental role of breakfast consumption in ensuring adequate total energy daily intake, the prevalence of skipping breakfast in Korea has increased over the past decade, mirroring similar trends observed in the USA^(24)^. Additionally, in the era of globalisation, economic growth may also impact dietary transitions in countries, leading to changes in the overall food environment and eating patterns^(25)^.

Our analysis revealed a significant increase in total fat intake across all age, sex and obesity status subgroups. Notably, participants in the younger age groups exhibited a more rapid increase in fat consumption compared with those in the older age group (15–18 years). Previous studies examining dietary fat intake in children identified lower levels of total fat and SFA intake in children from Asian countries than in those from Western countries^(26–28)^. However, Asian children have shown increasing trends in dietary fat consumption over time, reflecting transitions in dietary patterns from a traditional plant-based diet to a higher intake of meat and dairy products^(3)^. According to the dietary reference intake for Koreans in 2020, the proportion of fat intake (%) in Korean children and adolescents did not exceed 15–30 %, which corresponds to the findings of this study^(29)^. However, compared with the reference level of SFA intake (%), the study population’s consumption of SFA exceeded the upper limit of 7–8 % in all age and sex subgroups^(29)^, which is concerning as high intake of SFA is associated with increased CVD risk^(30)^. Therefore, appropriate fat consumption should be emphasised based on the dietary reference to prevent long-term complications.

Despite the increasing trends in energy intake from the five different fat subtypes during 2016–2020, the results varied when analysed by sex. Notably, N3 consumption increased more rapidly in boys than in girls, and the observed findings were not significant in girls. These findings align with recent reviews where children from different countries consistently have N3 intake levels below the dietary recommendations^(31,32)^. Many health benefits of N3 have been demonstrated, and their positive effects on CVD are widely recognised in adults^(33)^. In addition, a recent study on the role of daily N3 supplementation reported that it is likely to contribute to improved cognitive functioning in children and adolescents^(34,35)^. Our findings suggest that efforts to raise awareness of healthy fat choices, particularly among Korean girls, are essential to achieve a more balanced approach to the consumption of fat and reduce the burden of chronic disease.

While the overall energy intake from carbohydrates (%) declined, the proportion of energy from fat (%) increased, indicating a need for proportional adjustments in dietary macronutrient consumption. Future studies are warranted to determine the optimal dietary macronutrient ratio among Korean children and adolescents. Additionally, although total sugar intake (%) appeared to be declining overall, subgroup analysis revealed that this decreasing trend was limited to the 6–8-year age group. Given the adverse health effects of excessive sugar consumption, including obesity, type 2 diabetes, CVD and cancer^(36)^, efforts to reduce sugar intake should be emphasised through nutritional education, particularly among Korean children and adolescents aged 9–18 years. Furthermore, dietary fibre consumption in this study (18·1–21·6 g/d in boys and 15·6–18·4 g/d in girls) was below the recommended levels based on the 2020 dietary reference intake for Koreans, in which 25–30 g/d for boys and 20–25 g/d for girls are recommended^(29)^. Research has shown sufficient dietary fibre intake facilitates better bowel movement and prevents a rapid increase in blood glucose after meals^(37,38)^, highlighting the need for nutrition interventions to encourage a diet rich in fibre.

Anthropometric measurements in our study showed increasing trends in BMI, WC and the percentage of general obesity (defined by weight and BMI) as well as abdominal obesity over the 10-year period. Despite the observed decrease in total energy intake, the prevalence of obesity has continued to rise in line with the increasing consumption of fat. A notable finding in this study is that all participants with obesity consumed a higher percentage of fat (%) compared with those without obesity. While providing children with adequate dietary fat is essential for healthy growth and development, excessive consumption of SFA is suspected to be a contributing factor to childhood obesity^(34,39)^. These findings may be related to the increased consumption of fast foods in recent decades. Previous research based on a US national survey indicated that children and adolescents with frequent fast food intake consumed more total energy and fat^(6)^. Excessive intake of SFA is also associated with hypercholesterolemia, increasing the risk of CVD^(40)^. Given that fast foods are rich sources of SFA, their consumption can negatively impact the body composition and nutritional status of children and adolescents, contributing to the rising prevalence of childhood obesity^(40)^.

This study has certain limitations. First, the reliance on participants’ self-reported 24-h dietary recall may introduce recall bias. Additionally, dietary surveys completed by primary caregivers at home, without a specific age limit, might not accurately represent the dietary intake occurring outside the home environment. Future studies could benefit from incorporating school meal plans to collect more comprehensive daily intake data, particularly for school-age children and adolescents. Second, as this study specifically focused on Korean children and adolescents, our findings might not be generalisable to populations with different cultural contexts and dietary patterns. Third, the increasing trends in BMI and obesity were observed despite the decline in total energy intake. This might be due to poor diet quality characterised by excessive SFA intake and changes in lifestyle factors such as decreased physical activity and increased skipping of breakfast among Korean youth^(41,42)^. Lastly, the utilisation of dietary nutrient intake data without considering food intake in practice may limit the comprehensive understanding of people’s eating habits. Future research could explore trend analysis according to the food groups to provide a more holistic view.

Despite these limitations, our study has certain strengths. By using data from the KNHANES, a large-scale nationally representative survey in Korea, our findings can be more broadly applied to the young Korean population. Furthermore, the analysis covered a decade’s worth of data, which facilitated the evaluation of changes in macronutrient intake patterns over time. This cross-sectional approach yields critical insights into the dietary nutrient consumption trends among Korean children and adolescents. Finally, conducting subgroup analysis by age, sex and obesity status allowed for a deeper understanding of the factors influencing consumption trends. This enables paediatric nutritionists and healthcare practitioners to explore group-specific variations and to develop strategies targeted for the subgroups.

In conclusion, this population-based study, utilising the KNHANES data, provided valuable insights into the dietary macronutrient intake trends among Korean children and adolescents over a decade. The observed trends in dietary macronutrient consumption may have implications for future increases in adulthood obesity and related co-morbid conditions over the long term. Therefore, our findings underscore the importance of continuously monitoring dietary changes and developing health strategies to prevent the potential risk of diet-related non-communicable diseases. Considering the practicality of nutrition education, adopting food-based educational approaches to improve current nutrient intake may prove more effective in real-world settings. Efforts to facilitate more balanced dietary choices are warranted to support Korean children and adolescents’ healthier growth and development.

## Supporting information

Sung et al. supplementary material Sung et al. supplementary material

## Supplementary material

For supplementary material accompanying this paper visit https://doi.org/10.1017/S1368980024002180
