# Supplementary material for: Secular trends in dietary energy, carbohydrate, protein and fat intake among Korean children and adolescents
Source: Public Health Nutr. 2024 Nov 22;27(1):e251. doi: 10.1017/S1368980024002180 (PMC13740162; doi:10.1017/S1368980024002180)
Supplement: Supplementary file 1 [file S1368980024002180sup001.docx]

**Supporting Information**

Figure S1. Trends in dietary fiber intake according to (A) age and (B) sex subgroups (n = 7,972) *

Table S1. Trends in total energy intake and dietary nutrition consumption from 2010 to 2020*†

Table S2. Trends in total energy and macronutrient intake according to age, sex, and obesity status*†

Table S3. Trends in energy intake from total sugars. dietary fiber, and fat subtypes according to age, sex, and obesity status*†

**Figure S1. Trends in dietary fiber intake according to (A) age and (B) sex subgroups (n = 7,972) ***

*Survey-weighted linear regression was used to obtain beta coefficients and p-values. Rao-Scott chi-square test was used to obtain p-values for difference and interaction between groups.

**Table S1. Trends in total energy intake and dietary nutrition consumption from 2010 to 2020*†**

| **Characteristic** | **2010** | **2011** | **2012** | **2013** | **2014** | **2015** | **2016** | **2017** | **2018** | **2019** | **2020** | **coefficient** | **p-value** |
| --- | --- | --- | --- | --- | --- | --- | --- | --- | --- | --- | --- | --- | --- |
| Energy (kcal) | 2065.3 (28.7) | 2049.2 (27.3) | 2043.7 (36.3) | 2033.9 (25.0) | 2049.0 (31.9) | 2074.1 (29.0) | 2028.0 (31.5) | 2014.5 (37.2) | 1946.8 (34.4) | 1917.4 (28.7) | 1842.7 (30.6) | -17.25 | <0.001 |
| Carbohydrate (g) | 318.9 (4.4) | 320.6 (4.4) | 318.6 (5.4) | 309.3 (3.8) | 307.5 (5.0) | 308.9 (4.4) | 306.1 (4.8) | 302.8 (6.3) | 288.3 (5.5) | 280.2 (4.3) | 267.9 (4.3) | -4.63 | <0.001 |
| Protein (g) | 72.8 (1.3) | 73.4 (1.4) | 74.2 (1.9) | 70.9 (1.3) | 72.8 (1.4) | 73.6 (1.4) | 73.9 (1.5) | 72.1 (1.7) | 69.8 (1.6) | 71.3 (1.3) | 68.1 (1.5) | -0.36 | 0.010 |
| Fat (g) | 54.9 (1.3) | 52.6 (1.1) | 53.1 (1.6) | 53.9 (1.0) | 55.7 (1.3) | 57.4 (1.3) | 54.2 (1.4) | 55.2 (1.4) | 54.6 (1.5) | 55.0 (1.3) | 53.1 (1.5) | 0.08 | 0.556 |
| SFA (g) | - | - | - | 17.5 (0.3) | 18.3 (0.5) | 18.7 (0.4) | 19.1 (0.5) | 19.5 (0.5) | 19.7 (0.6) | 19.5 (0.5) | 18.3 (0.6) | 0.19 | 0.012 |
| MUFA (g) | - | - | - | 17.5 (0.4) | 18.2 (0.5) | 18.9 (0.5) | 17.2 (0.6) | 17.4 (0.5) | 18.1 (0.6) | 18.3 (0.5) | 18.0 (0.6) | 0.03 | 0.731 |
| PUFA (g) | - | - | - | 11.7 (0.3) | 11.9 (0.3) | 12.1 (0.3) | 12.2 (0.3) | 12.7 (0.4) | 12.0 (0.3) | 12.0 (0.3) | 12.1 (0.4) | 0.05 | 0.315 |
| N3 (g) | - | - | - | 1.4 (0.0) | 1.4 (0.0) | 1.5 (0.0) | 1.5 (0.1) | 1.6 (0.1) | 1.5 (0.1) | 1.5 (0.1) | 1.4 (0.1) | 0.01 | 0.271 |
| N6 (g) | - | - | - | 10.4 (0.3) | 10.5 (0.3) | 10.7 (0.3) | 10.7 (0.3) | 11.0 (0.3) | 10.4 (0.3) | 10.5 (0.3) | 10.6 (0.3) | 0.02 | 0.729 |
| Cholesterol (mg) | - | - | - | 288.1 (8.9) | 279.8 (8.4) | 299.7 (9.8) | 273.9 (8.1) | 263.6 (8.3) | 260.5 (10.9) | 277.8 (6.4) | 271.6 (8.0) | -3.05 | 0.021 |
| Dietary fiber (g) | - | - | - | 17.3 (0.4) | 17.6 (0.5) | 17.3 (0.4) | 20.2 (0.5) | 19.8 (0.6) | 18.1 (0.5) | 18.2 (0.4) | 17.7 (0.4) | 0.12 | 0.066 |
| Total sugars (g) | - | - | - | - | - | - | 72.7 (1.8) | 72.1 (2.2) | 65.9 (1.8) | 67.2 (1.7) | 62.1 (2.0) | -2.60 | <0.001 |

SFA, saturated fatty acids; MUFA, monounsaturated fatty acids; PUFA, polyunsaturated fatty acids; N3, omega-3 fatty acids; N6, omega-6 fatty acids

*Survey-weighted linear regression was used to obtain beta coefficients and p-values.

†n = 11,816 for energy, carbohydrate, protein, and fat / n = 7,972 for fat subtypes and dietary fiber / n = 4,738 for total sugars

**Table S2. Trends in total energy and macronutrient intake according to age, sex, and obesity status*†**

| **Subgroup** | | **Nutritions** | **2010** | **2011** | **2012** | **2013** | **2014** | **2015** | **2016** | **2017** | **2018** | **2019** | **2020** | **Coefficient** | **p-value** |
| --- | --- | --- | --- | --- | --- | --- | --- | --- | --- | --- | --- | --- | --- | --- | --- |
| **Age** | **6-8** | **Energy (kcal)** | 1684.4 (36.9) | 1755.7 (34.4) | 1650.3 (47.0) | 1673.9 (35.5) | 1746.5 (36.7) | 1774.2 (41.9) | 1732.6 (48.6) | 1693.6 (40.2) | 1626.4 (36.8) | 1624.1 (33.5) | 1675.0 (52.6) | -5.83 | 0.140 |
|  |  | **Carbohydrate (%)** | 65.0 (0.6) | 64.2 (0.7) | 65.1 (0.7) | 63.8 (0.6) | 62.5 (0.7) | 62.4 (0.8) | 63.6 (0.6) | 62.2 (0.6) | 61.8 (0.7) | 60.1 (0.6) | 60.9 (0.6) | -0.44 | <0.001 |
|  |  | **Protein (%)** | 13.9 (0.2) | 13.8 (0.2) | 13.6 (0.3) | 13.4 (0.2) | 13.9 (0.3) | 14.0 (0.3) | 13.6 (0.2) | 14.0 (0.3) | 13.8 (0.3) | 14.4 (0.2) | 14.6 (0.3) | 0.07 | 0.005 |
|  |  | **Fat (%)** | 21.1 (0.5) | 22.0 (0.5) | 21.4 (0.5) | 22.9 (0.5) | 23.6 (0.6) | 23.7 (0.6) | 22.8 (0.5) | 23.7 (0.5) | 24.4 (0.6) | 25.5 (0.5) | 24.6 (0.5) | 0.37 | <0.001 |
|  | **9-11** | **Energy (kcal)** | 1917.1 (33.5) | 1946.2 (44.0) | 1971.4 (56.3) | 2007.5 (43.1) | 2047.7 (54.3) | 2055.4 (63.8) | 2012.4 (49.7) | 2006.9 (55.4) | 1843.4 (50.9) | 1928.0 (43.4) | 1724.4 (52.4) | -11.26 | 0.012 |
|  |  | **Carbohydrate (%)** | 64.2 (0.5) | 65.2 (0.5) | 63.3 (0.7) | 62.6 (0.7) | 61.4 (0.7) | 61.0 (0.7) | 63.0 (0.7) | 61.4 (0.6) | 61.2 (0.7) | 60.0 (0.6) | 59.4 (0.8) | -0.47 | <0.001 |
|  |  | **Protein (%)** | 13.9 (0.2) | 13.9 (0.2) | 14.1 (0.3) | 14.1 (0.4) | 14.4 (0.4) | 14.3 (0.3) | 14.0 (0.2) | 14.4 (0.2) | 14.2 (0.2) | 15.0 (0.3) | 15.0 (0.4) | 0.09 | <0.001 |
|  |  | **Fat (%)** | 21.9 (0.4) | 20.9 (0.4) | 22.6 (0.6) | 23.3 (0.5) | 24.2 (0.6) | 24.6 (0.6) | 23.0 (0.6) | 24.2 (0.5) | 24.6 (0.6) | 25.0 (0.6) | 25.6 (0.7) | 0.38 | <0.001 |
|  | **12-14** | **Energy (kcal)** | 2221.6 (53.9) | 2117.1 (51.7) | 2108.3 (54.9) | 2120.0 (49.7) | 2210.5 (63.2) | 2147.8 (53.0) | 2104.8 (55.3) | 2132.0 (63.9) | 2062.6 (63.5) | 2117.3 (61.0) | 1969.0 (56.4) | -12.95 | 0.016 |
|  |  | **Carbohydrate (%)** | 63.2 (0.7) | 63.3 (0.8) | 64.2 (0.8) | 63.2 (0.7) | 61.2 (0.6) | 61.2 (0.7) | 61.2 (0.7) | 60.6 (1.0) | 61.2 (0.7) | 58.9 (0.9) | 59.4 (0.7) | -0.46 | <0.001 |
|  |  | **Protein (%)** | 14.1 (0.2) | 14.2 (0.2) | 14.3 (0.3) | 13.7 (0.3) | 14.2 (0.3) | 14.4 (0.3) | 15.0 (0.3) | 14.8 (0.3) | 14.1 (0.2) | 14.9 (0.3) | 15.2 (0.3) | 0.09 | <0.001 |
|  |  | **Fat (%)** | 22.7 (0.6) | 22.5 (0.6) | 21.5 (0.7) | 23.1 (0.6) | 24.6 (0.5) | 24.4 (0.6) | 23.8 (0.6) | 24.7 (0.8) | 24.6 (0.6) | 26.3 (0.8) | 25.5 (0.7) | 0.37 | <0.001 |
|  | **15-18** | **Energy (kcal)** | 2203.6 (69.9) | 2256.4 (59.3) | 2216.6 (69.4) | 2204.3 (57.1) | 2117.3 (70.2) | 2202.4 (63.2) | 2148.8 (57.7) | 2133.8 (69.2) | 2137.8 (69.5) | 2002.1 (63.6) | 1926.0 (60.8) | -23.88 | <0.001 |
|  |  | **Carbohydrate (%)** | 61.5 (0.8) | 61.6 (0.8) | 62.2 (0.9) | 61.5 (0.6) | 60.3 (0.8) | 60.9 (0.7) | 60.8 (0.8) | 60.8 (0.7) | 59.2 (1.0) | 59.7 (0.8) | 58.6 (0.8) | -0.28 | <0.001 |
|  |  | **Protein (%)** | 14.1 (0.3) | 14.6 (0.3) | 15.0 (0.4) | 14.6 (0.3) | 14.8 (0.4) | 14.4 (0.3) | 15.4 (0.3) | 14.7 (0.3) | 15.2 (0.3) | 15.5 (0.3) | 15.1 (0.4) | 0.09 | 0.005 |
|  |  | **Fat (%)** | 24.4 (0.7) | 23.8 (0.6) | 22.8 (0.7) | 23.9 (0.5) | 24.8 (0.7) | 24.8 (0.6) | 23.8 (0.6) | 24.5 (0.6) | 25.5 (0.9) | 24.8 (0.7) | 26.3 (0.6) | 0.19 | 0.004 |
| **Sex** | **Male** | **Energy (kcal)** | 2226.1 (44.2) | 2266.8 (42.0) | 2218.2 (51.6) | 2251.0 (39.3) | 2259.3 (49.5) | 2286.1 (42.8) | 2248.0 (44.7) | 2234.7 (50.3) | 2144.0 (49.3) | 2094.7 (40.5) | 1998.7 (44.9) | -17.66 | <0.001 |
|  |  | **Carbohydrate (%)** | 62.8 (0.5) | 63.0 (0.6) | 63.5 (0.5) | 62.5 (0.4) | 60.9 (0.6) | 60.4 (0.5) | 61.6 (0.5) | 60.5 (0.6) | 61.1 (0.6) | 58.9 (0.5) | 59.3 (0.6) | -0.41 | <0.001 |
|  |  | **Protein (%)** | 14.3 (0.2) | 14.3 (0.2) | 14.4 (0.2) | 14.2 (0.2) | 14.6 (0.2) | 14.5 (0.2) | 14.9 (0.2) | 14.7 (0.2) | 14.7 (0.2) | 15.4 (0.2) | 15.2 (0.3) | 0.10 | <0.001 |
|  |  | **Fat (%)** | 22.9 (0.4) | 22.7 (0.5) | 22.0 (0.5) | 23.3 (0.4) | 24.6 (0.5) | 25.1 (0.4) | 23.5 (0.4) | 24.8 (0.5) | 24.2 (0.5) | 25.6 (0.4) | 25.5 (0.5) | 0.31 | <0.001 |
|  | **Female** | **Energy (kcal)** | 1832.5 (36.9) | 1825.8 (32.5) | 1831.7 (45.9) | 1816.5 (38.2) | 1829.5 (35.5) | 1832.3 (38.5) | 1780.7 (34.2) | 1772.8 (39.8) | 1743.9 (41.5) | 1740.4 (37.6) | 1642.7 (39.7) | -14.73 | <0.001 |
|  |  | **Carbohydrate (%)** | 63.6 (0.6) | 63.5 (0.5) | 63.3 (0.7) | 62.6 (0.5) | 61.5 (0.6) | 62.3 (0.6) | 62.2 (0.6) | 61.9 (0.5) | 60.2 (0.7) | 60.5 (0.5) | 59.7 (0.7) | -0.38 | <0.001 |
|  |  | **Protein (%)** | 13.7 (0.2) | 14.1 (0.2) | 14.3 (0.3) | 13.9 (0.2) | 14.3 (0.3) | 14.0 (0.2) | 14.4 (0.2) | 14.3 (0.2) | 14.3 (0.2) | 14.6 (0.2) | 14.7 (0.3) | 0.07 | 0.001 |
|  |  | **Fat (%)** | 22.7 (0.5) | 22.4 (0.4) | 22.4 (0.6) | 23.5 (0.4) | 24.2 (0.5) | 23.7 (0.4) | 23.4 (0.4) | 23.8 (0.4) | 25.5 (0.6) | 25.0 (0.5) | 25.6 (0.6) | 0.31 | <0.001 |
| **Abdominal obesity‡** | **Obesity** | **Energy (kcal)** | 2192.7 (175.3) | 2182.8 (167.7) | 2163.9 (207.8) | 1899.3 (123.3) | 2660.5 (359.2) | 2321.0 (171.8) | 2285.8 (155.6) | 1869.2 (135.0) | 2271.6 (191.0) | 2078.3 (138.4) | 2084.6 (140.9) | -12.68 | 0.427 |
|  |  | **Carbohydrate (%)** | 58.8 (2.0) | 64.6 (1.7) | 66.7 (2.4) | 57.4 (2.0) | 60.9 (1.9) | 60.9 (2.4) | 59.6 (2.2) | 62.1 (1.7) | 62.3 (1.9) | 60.2 (1.4) | 57.0 (1.4) | -0.34 | 0.061 |
|  |  | **Protein (%)** | 15.6 (0.6) | 15.0 (0.7) | 14.7 (0.8) | 17.5 (1.3) | 13.8 (0.7) | 14.9 (0.8) | 16.3 (1.0) | 14.1 (0.6) | 14.4 (0.8) | 15.8 (0.5) | 16.5 (0.6) | 0.05 | 0.465 |
|  |  | **Fat (%)** | 25.6 (1.8) | 20.5 (1.3) | 18.6 (2.0) | 25.1 (1.4) | 25.3 (2.0) | 24.2 (1.8) | 24.1 (1.7) | 23.8 (1.5) | 23.2 (1.7) | 24.0 (1.3) | 26.5 (1.2) | 0.29 | 0.059 |
|  | **Normal** | **Energy (kcal)** | 2038.1 (30.8) | 2055.6 (28.4) | 2037.2 (37.6) | 2048.3 (29.2) | 2026.7 (31.7) | 2056.2 (30.6) | 2007.9 (31.9) | 2019.6 (36.1) | 1928.7 (32.9) | 1910.6 (28.8) | 1811.2 (32.3) | -18.14 | <0.001 |
|  |  | **Carbohydrate (%)** | 63.3 (0.4) | 63.2 (0.4) | 63.3 (0.5) | 62.7 (0.3) | 61.2 (0.4) | 61.3 (0.4) | 62.1 (0.4) | 61.1 (0.5) | 60.6 (0.5) | 59.7 (0.4) | 59.7 (0.5) | -0.39 | <0.001 |
|  |  | **Protein (%)** | 14.0 (0.1) | 14.2 (0.2) | 14.4 (0.2) | 14.0 (0.2) | 14.4 (0.2) | 14.2 (0.2) | 14.5 (0.2) | 14.5 (0.2) | 14.5 (0.2) | 15.0 (0.2) | 14.9 (0.2) | 0.08 | <0.001 |
|  |  | **Fat (%)** | 22.7 (0.3) | 22.6 (0.3) | 22.3 (0.4) | 23.3 (0.3) | 24.4 (0.4) | 24.4 (0.3) | 23.4 (0.3) | 24.3 (0.4) | 25.0 (0.4) | 25.4 (0.3) | 25.5 (0.4) | 0.31 | <0.001 |
| **Obesity (weight)§** | **Obese** | **Energy (kcal)** | 1829.3 (106.0) | 1982.5 (139.6) | 1870.9 (201.0) | 1919.1 (109.8) | 2280.4 (218.0) | 2071.2 (124.7) | 2089.1 (129.8) | 2104.4 (136.7) | 1994.2 (145.4) | 2067.1 (120.9) | 1967.5 (122.0) | 10.58 | 0.404 |
|  |  | **Carbohydrate (%)** | 61.5 (1.2) | 64.6 (1.9) | 65.9 (2.8) | 61.4 (1.7) | 60.5 (1.4) | 61.4 (2.1) | 58.2 (1.7) | 62.7 (1.6) | 62.0 (1.3) | 60.3 (1.6) | 57.3 (1.2) | -0.46 | 0.003 |
|  |  | **Protein (%)** | 15.1 (0.6) | 14.8 (0.7) | 14.3 (0.7) | 15.6 (1.0) | 15.2 (1.0) | 15.0 (0.8) | 16.5 (1.0) | 13.4 (0.5) | 14.8 (0.7) | 15.3 (0.5) | 15.2 (0.5) | 0.01 | 0.868 |
|  |  | **Fat (%)** | 23.4 (1.0) | 20.6 (1.5) | 19.8 (2.3) | 23.1 (1.2) | 24.3 (1.4) | 23.7 (1.5) | 25.3 (1.6) | 23.9 (1.4) | 23.2 (1.1) | 24.4 (1.5) | 27.5 (1.0) | 0.45 | <0.001 |
|  | **Normal** | **Energy (kcal)** | 2056.7 (31.2) | 2064.1 (28.8) | 2046.2 (38.1) | 2047.7 (29.2) | 2037.3 (32.6) | 2071.3 (31.0) | 2018.8 (32.5) | 2006.1 (37.3) | 1942.3 (32.8) | 1908.9 (28.0) | 1820.1 (32.0) | -19.09 | <0.001 |
|  |  | **Carbohydrate (%)** | 63.2 (0.4) | 63.2 (0.4) | 63.3 (0.5) | 62.6 (0.3) | 61.2 (0.4) | 61.3 (0.4) | 62.1 (0.4) | 61.1 (0.5) | 60.6 (0.5) | 59.7 (0.4) | 59.7 (0.4) | -0.38 | <0.001 |
|  |  | **Protein (%)** | 14.0 (0.1) | 14.2 (0.2) | 14.4 (0.2) | 14.0 (0.2) | 14.4 (0.2) | 14.2 (0.2) | 14.5 (0.2) | 14.6 (0.2) | 14.4 (0.2) | 15.0 (0.2) | 15.0 (0.2) | 0.09 | <0.001 |
|  |  | **Fat (%)** | 22.8 (0.4) | 22.6 (0.3) | 22.3 (0.4) | 23.4 (0.3) | 24.4 (0.4) | 24.5 (0.4) | 23.3 (0.3) | 24.3 (0.4) | 24.9 (0.4) | 25.3 (0.3) | 25.4 (0.4) | 0.30 | <0.001 |
| **Obesity (BMI)¶** | **Obese** | **Energy (kcal)** | 1809.9 (104.6) | 1846.9 (129.6) | 1856.1 (203.1) | 1848.6 (96.6) | 2456.8 (237.4) | 2003.4 (113.8) | 2030.5 (135.5) | 1935.4 (148.1) | 1887.1 (137.1) | 1947.3 (113.5) | 1939.8 (106.3) | 7.28 | 0.530 |
|  |  | **Carbohydrate (%)** | 63.1 (1.4) | 65.5 (1.8) | 62.0 (2.4) | 62.4 (1.5) | 61.3 (1.6) | 62.0 (2.1) | 58.5 (1.8) | 62.4 (1.6) | 62.5 (1.3) | 60.9 (1.4) | 58.3 (1.2) | -0.45 | 0.002 |
|  |  | **Protein (%)** | 14.8 (0.5) | 14.7 (0.7) | 14.7 (0.7) | 14.4 (0.5) | 14.4 (0.8) | 14.8 (0.8) | 16.4 (1.0) | 13.6 (0.6) | 14.3 (0.6) | 15.1 (0.6) | 15.3 (0.6) | 0.04 | 0.529 |
|  |  | **Fat (%)** | 22.2 (1.1) | 19.8 (1.4) | 23.3 (2.0) | 23.2 (1.2) | 24.2 (1.6) | 23.2 (1.5) | 25.2 (1.7) | 24.0 (1.3) | 23.2 (1.1) | 24.0 (1.3) | 26.5 (1.0) | 0.41 | <0.001 |
|  | **Normal** | **Energy (kcal)** | 2059.6 (31.1) | 2071.4 (28.8) | 2045.3 (39.0) | 2051.4 (29.3) | 2031.7 (32.1) | 2075.6 (30.8) | 2022.2 (32.4) | 2016.0 (37.2) | 1950.1 (34.0) | 1918.8 (28.7) | 1821.9 (32.1) | -18.61 | <0.001 |
|  |  | **Carbohydrate (%)** | 63.2 (0.4) | 63.2 (0.4) | 63.5 (0.5) | 62.5 (0.3) | 61.2 (0.4) | 61.3 (0.4) | 62.1 (0.4) | 61.1 (0.5) | 60.6 (0.5) | 59.7 (0.4) | 59.6 (0.5) | -0.38 | <0.001 |
|  |  | **Protein (%)** | 14.0 (0.1) | 14.2 (0.2) | 14.4 (0.2) | 14.1 (0.2) | 14.4 (0.2) | 14.2 (0.2) | 14.5 (0.2) | 14.5 (0.2) | 14.5 (0.2) | 15.0 (0.2) | 15.0 (0.2) | 0.09 | <0.001 |
|  |  | **Fat (%)** | 22.8 (0.4) | 22.7 (0.3) | 22.2 (0.4) | 23.4 (0.3) | 24.4 (0.4) | 24.5 (0.3) | 23.3 (0.3) | 24.3 (0.4) | 25.0 (0.4) | 25.3 (0.4) | 25.4 (0.4) | 0.30 | <0.001 |

SFA, saturated fatty acids; MUFA, monounsaturated fatty acids; PUFA, polyunsaturated fatty acids; N3, omega-3 fatty acids; N6, omega-6 fatty acids

*Survey-weighted linear regression was used to obtain beta coefficients and p-values.

†n = 11,816 for energy, carbohydrate, protein, and fat / n = 7,972 for fat subtypes and dietary fiber / n = 4,429 for total sugars

‡ n = 11,047 for energy, carbohydrate, protein, and fat / n = 7,435 for fat subtypes and dietary fiber / n = 4,738 for total sugars

§ n = 11,065 for energy, carbohydrate, protein, and fat / n = 7,446 for fat subtypes and dietary fiber / n = 4,438 for total sugars

¶ n = 11,063 for energy, carbohydrate, protein, and fat / n = 7,444 for fat subtypes and dietary fiber / n = 4,436 for total sugars

**Table S3. Trends in energy intake from total sugars, dietary fiber, and fat subtypes according to age, sex, and obesity status*†**

| **Subgroup** | | **Nutritions** | **2013** | **2014** | **2015** | **2016** | **2017** | **2018** | **2019** | **2020** | **Coefficient** | **p-value** |
| --- | --- | --- | --- | --- | --- | --- | --- | --- | --- | --- | --- | --- |
| **Age** | **6-8** | Total sugars (%) | - | - | - | 15.2 (0.4) | 15.7 (0.5) | 15.1 (0.6) | 14.9 (0.4) | 13.5 (0.6) | -0.42 | 0.010 |
|  |  | Dietary fiber (g/day) | 14.1 (0.5) | 16.1 (0.6) | 14.9 (0.6) | 17.2 (0.6) | 17.2 (0.8) | 14.5 (0.6) | 15.0 (0.4) | 15.4 (0.7) | 0.02 | 0.828 |
|  |  | Saturated fatty acid (%) | 7.9 (0.2) | 8.0 (0.2) | 7.9 (0.2) | 8.3 (0.2) | 8.6 (0.2) | 9.2 (0.3) | 9.5 (0.2) | 8.6 (0.3) | 0.20 | <0.001 |
|  |  | MUFA (%) | 7.2 (0.2) | 7.7 (0.2) | 7.6 (0.2) | 7.3 (0.2) | 7.4 (0.2) | 7.8 (0.2) | 8.1 (0.2) | 8.2 (0.3) | 0.11 | 0.002 |
|  |  | PUFA (%) | 4.6 (0.2) | 4.7 (0.1) | 4.8 (0.2) | 4.9 (0.1) | 5.4 (0.2) | 5.0 (0.2) | 5.4 (0.2) | 5.3 (0.2) | 0.11 | <0.001 |
|  |  | N3 (%) | 0.6 (0.0) | 0.6 (0.0) | 0.6 (0.0) | 0.6 (0.0) | 0.8 (0.0) | 0.7 (0.0) | 0.7 (0.0) | 0.7 (0.0) | 0.01 | 0.030 |
|  |  | N6 (%) | 4.0 (0.1) | 4.1 (0.1) | 4.2 (0.2) | 4.3 (0.1) | 4.6 (0.2) | 4.3 (0.2) | 4.6 (0.1) | 4.6 (0.1) | 0.09 | <0.001 |
|  |  | Cholesterol (mg/day) | 233.5 (15.2) | 233.1 (13.9) | 284.9 (15.2) | 235.1 (11.8) | 229.3 (10.5) | 221.0 (13.1) | 240.8 (13.1) | 253.0 (12.0) | -0.24 | 0.911 |
|  | **9-11** | Total sugars (%) | - | - | - | 14.7 (0.5) | 14.9 (0.6) | 13.9 (0.4) | 14.0 (0.4) | 13.8 (0.8) | -0.26 | 0.176 |
|  |  | Dietary fiber (g/day) | 17.5 (0.6) | 17.1 (0.7) | 17.3 (0.6) | 19.7 (0.7) | 19.7 (0.8) | 18.0 (0.6) | 17.9 (0.6) | 16.8 (0.8) | 0.04 | 0.744 |
|  |  | Saturated fatty acid (%) | 7.9 (0.2) | 8.2 (0.2) | 8.1 (0.2) | 8.3 (0.3) | 8.8 (0.2) | 9.0 (0.3) | 8.8 (0.3) | 8.9 (0.3) | 0.16 | <0.001 |
|  |  | MUFA (%) | 7.4 (0.2) | 7.7 (0.2) | 8.0 (0.2) | 7.2 (0.2) | 7.6 (0.2) | 8.1 (0.3) | 8.1 (0.2) | 8.6 (0.3) | 0.12 | 0.002 |
|  |  | PUFA (%) | 4.8 (0.2) | 4.8 (0.2) | 4.9 (0.2) | 5.0 (0.2) | 5.2 (0.2) | 5.2 (0.1) | 5.5 (0.2) | 5.6 (0.2) | 0.12 | <0.001 |
|  |  | N3 (%) | 0.6 (0.0) | 0.6 (0.0) | 0.6 (0.0) | 0.6 (0.0) | 0.7 (0.0) | 0.6 (0.0) | 0.7 (0.0) | 0.7 (0.0) | 0.02 | 0.003 |
|  |  | N6 (%) | 4.2 (0.2) | 4.3 (0.2) | 4.3 (0.2) | 4.3 (0.1) | 4.5 (0.1) | 4.5 (0.1) | 4.8 (0.2) | 4.9 (0.2) | 0.09 | <0.001 |
|  |  | Cholesterol (mg/day) | 278.0 (14.0) | 313.2 (20.9) | 310.8 (21.2) | 272.4 (14.8) | 270.5 (14.1) | 263.0 (14.0) | 290.1 (13.8) | 258.8 (14.5) | -4.45 | 0.069 |
|  | **12-14** | Total sugars (%) | - | - | - | 13.6 (0.5) | 14.3 (0.7) | 13.3 (0.5) | 13.7 (0.8) | 13.3 (0.5) | -0.12 | 0.504 |
|  |  | Dietary fiber (g/day) | 17.8 (0.7) | 19.3 (0.9) | 17.9 (0.7) | 21.6 (0.9) | 20.8 (1.0) | 19.3 (0.9) | 20.6 (0.9) | 19.8 (0.9) | 0.30 | 0.019 |
|  |  | Saturated fatty acid (%) | 7.5 (0.2) | 7.9 (0.2) | 8.0 (0.2) | 8.3 (0.3) | 8.4 (0.3) | 8.5 (0.3) | 9.1 (0.3) | 8.6 (0.3) | 0.19 | <0.001 |
|  |  | MUFA (%) | 7.3 (0.2) | 7.9 (0.2) | 8.0 (0.2) | 7.3 (0.3) | 7.8 (0.3) | 8.0 (0.2) | 8.8 (0.3) | 8.5 (0.3) | 0.15 | <0.001 |
|  |  | PUFA (%) | 4.9 (0.2) | 5.4 (0.2) | 5.0 (0.2) | 5.4 (0.2) | 5.7 (0.2) | 5.7 (0.2) | 5.7 (0.2) | 5.9 (0.2) | 0.14 | <0.001 |
|  |  | N3 (%) | 0.6 (0.0) | 0.6 (0.0) | 0.6 (0.0) | 0.7 (0.0) | 0.8 (0.1) | 0.7 (0.1) | 0.7 (0.1) | 0.7 (0.0) | 0.02 | 0.003 |
|  |  | N6 (%) | 4.3 (0.2) | 4.8 (0.2) | 4.4 (0.2) | 4.7 (0.1) | 4.9 (0.2) | 5.0 (0.2) | 5.0 (0.2) | 5.2 (0.2) | 0.11 | <0.001 |
|  |  | Cholesterol (mg/day) | 306.7 (20.8) | 286.2 (18.6) | 311.1 (17.9) | 289.0 (13.8) | 303.5 (19.9) | 264.3 (16.3) | 304.2 (15.4) | 295.6 (16.4) | -1.42 | 0.614 |
|  | **15-18** | Total sugars (%) | - | - | - | 14.1 (0.5) | 13.2 (0.6) | 13.1 (0.5) | 13.6 (0.6) | 13.2 (0.6) | -0.15 | 0.395 |
|  |  | Dietary fiber (g/day) | 18.5 (0.7) | 18.0 (0.9) | 17.9 (0.7) | 20.9 (0.7) | 20.3 (0.9) | 20.0 (0.9) | 19.3 (0.8) | 18.0 (0.8) | 0.13 | 0.292 |
|  |  | Saturated fatty acid (%) | 7.4 (0.2) | 7.8 (0.3) | 7.8 (0.2) | 8.1 (0.3) | 8.6 (0.3) | 8.9 (0.4) | 8.6 (0.3) | 8.6 (0.2) | 0.20 | <0.001 |
|  |  | MUFA (%) | 7.7 (0.2) | 8.0 (0.3) | 8.0 (0.2) | 7.4 (0.3) | 7.5 (0.3) | 8.5 (0.4) | 8.3 (0.3) | 8.7 (0.3) | 0.11 | 0.005 |
|  |  | PUFA (%) | 5.2 (0.1) | 5.4 (0.2) | 5.3 (0.2) | 5.5 (0.2) | 5.7 (0.2) | 5.6 (0.2) | 5.4 (0.2) | 6.2 (0.3) | 0.09 | 0.003 |
|  |  | N3 (%) | 0.6 (0.0) | 0.6 (0.0) | 0.6 (0.0) | 0.7 (0.0) | 0.7 (0.0) | 0.7 (0.0) | 0.6 (0.0) | 0.7 (0.1) | 0.01 | 0.027 |
|  |  | N6 (%) | 4.6 (0.1) | 4.8 (0.2) | 4.7 (0.2) | 4.8 (0.2) | 5.0 (0.1) | 4.9 (0.1) | 4.8 (0.2) | 5.4 (0.2) | 0.07 | 0.012 |
|  |  | Cholesterol (mg/day) | 306.1 (15.3) | 287.7 (15.7) | 300.7 (19.6) | 283.9 (14.8) | 268.2 (14.5) | 291.6 (30.4) | 288.1 (13.4) | 275.3 (16.2) | -3.19 | 0.221 |
| **Sex** | **Male** | Total sugars (%) | - | - | - | 13.6 (0.3) | 13.7 (0.4) | 12.9 (0.4) | 12.7 (0.3) | 12.8 (0.5) | -0.24 | 0.055 |
|  |  | Dietary fiber (g/day) | 18.9 (0.5) | 19.2 (0.7) | 18.1 (0.5) | 21.6 (0.6) | 21.6 (0.8) | 19.9 (0.6) | 19.7 (0.6) | 19.0 (0.5) | 0.11 | 0.198 |
|  |  | Saturated fatty acid (%) | 7.5 (0.1) | 8.1 (0.2) | 8.1 (0.2) | 8.2 (0.2) | 8.7 (0.2) | 8.5 (0.2) | 9.0 (0.2) | 8.6 (0.2) | 0.16 | <0.001 |
|  |  | MUFA (%) | 7.5 (0.2) | 8.0 (0.2) | 8.1 (0.2) | 7.4 (0.2) | 7.7 (0.2) | 8.0 (0.2) | 8.5 (0.2) | 8.6 (0.2) | 0.12 | <0.001 |
|  |  | PUFA (%) | 4.9 (0.1) | 5.1 (0.1) | 5.2 (0.1) | 5.2 (0.1) | 5.6 (0.1) | 5.5 (0.1) | 5.6 (0.1) | 5.8 (0.2) | 0.12 | <0.001 |
|  |  | N3 (%) | 0.6 (0.0) | 0.6 (0.0) | 0.6 (0.0) | 0.7 (0.0) | 0.7 (0.0) | 0.7 (0.0) | 0.7 (0.0) | 0.7 (0.0) | 0.02 | <0.001 |
|  |  | N6 (%) | 4.3 (0.1) | 4.5 (0.1) | 4.6 (0.1) | 4.5 (0.1) | 4.9 (0.1) | 4.8 (0.1) | 4.9 (0.1) | 5.1 (0.2) | 0.09 | <0.001 |
|  |  | Cholesterol (mg/day) | 300.9 (11.7) | 305.8 (11.8) | 339.4 (13.9) | 299.1 (11.8) | 297.9 (11.8) | 288.5 (21.7) | 301.0 (8.7) | 282.7 (11.3) | -3.48 | 0.066 |
|  | **Female** | Total sugars (%) | - | - | - | 15.2 (0.4) | 15.0 (0.4) | 14.6 (0.4) | 15.4 (0.5) | 14.1 (0.5) | -0.18 | 0.203 |
|  |  | Dietary fiber (g/day) | 15.6 (0.5) | 16.2 (0.5) | 16.1 (0.4) | 18.4 (0.5) | 17.5 (0.6) | 16.6 (0.6) | 16.8 (0.5) | 15.8 (0.6) | 0.08 | 0.334 |
|  |  | Saturated fatty acid (%) | 7.7 (0.1) | 7.8 (0.2) | 7.7 (0.2) | 8.2 (0.2) | 8.5 (0.2) | 9.4 (0.3) | 9.0 (0.2) | 8.8 (0.2) | 0.22 | <0.001 |
|  |  | MUFA (%) | 7.5 (0.1) | 7.7 (0.2) | 7.7 (0.2) | 7.3 (0.2) | 7.4 (0.2) | 8.3 (0.3) | 8.1 (0.2) | 8.4 (0.2) | 0.12 | <0.001 |
|  |  | PUFA (%) | 4.9 (0.1) | 5.2 (0.2) | 4.9 (0.1) | 5.3 (0.1) | 5.4 (0.1) | 5.3 (0.1) | 5.4 (0.1) | 5.7 (0.2) | 0.09 | <0.001 |
|  |  | N3 (%) | 0.6 (0.0) | 0.6 (0.0) | 0.6 (0.0) | 0.7 (0.0) | 0.7 (0.0) | 0.7 (0.0) | 0.7 (0.0) | 0.7 (0.0) | 0.01 | 0.056 |
|  |  | N6 (%) | 4.4 (0.1) | 4.6 (0.1) | 4.3 (0.1) | 4.6 (0.1) | 4.6 (0.1) | 4.6 (0.1) | 4.7 (0.1) | 5.1 (0.2) | 0.07 | <0.001 |
|  |  | Cholesterol (mg/day) | 270.4 (11.9) | 258.4 (11.9) | 260.2 (11.7) | 243.2 (8.9) | 233.9 (9.5) | 239.8 (11.9) | 259.9 (10.2) | 257.4 (11.0) | -2.05 | 0.253 |
| **Abdominal**  **obesity‡** | **Obesity** | Total sugars (%) | - | - | - | 13.3 (1.1) | 13.4 (1.6) | 13.9 (1.4) | 12.8 (1.3) | 10.5 (0.6) | -0.64 | 0.048 |
|  |  | Dietary fiber (g/day) | 17.6 (2.0) | 21.9 (4.0) | 18.6 (1.5) | 24.7 (1.8) | 16.0 (1.4) | 24.6 (3.8) | 20.0 (1.9) | 18.0 (1.3) | -0.06 | 0.858 |
|  |  | Saturated fatty acid (%) | 7.8 (0.6) | 8.9 (1.2) | 7.2 (0.6) | 7.3 (0.6) | 8.2 (0.7) | 8.0 (0.7) | 8.4 (0.6) | 8.4 (0.6) | 0.09 | 0.398 |
|  |  | MUFA (%) | 7.9 (0.5) | 7.6 (0.6) | 7.5 (0.7) | 7.6 (0.7) | 7.6 (0.6) | 7.8 (0.7) | 8.1 (0.5) | 9.4 (0.6) | 0.19 | 0.037 |
|  |  | PUFA (%) | 5.7 (0.5) | 5.3 (0.5) | 5.3 (0.5) | 6.2 (0.4) | 5.5 (0.4) | 5.2 (0.4) | 5.1 (0.3) | 5.8 (0.4) | -0.01 | 0.871 |
|  |  | N3 (%) | 0.7 (0.1) | 0.7 (0.1) | 0.6 (0.1) | 0.8 (0.1) | 0.7 (0.1) | 0.6 (0.1) | 0.6 (0.1) | 0.8 (0.1) | 0.00 | 0.805 |
|  |  | N6 (%) | 5.0 (0.5) | 4.6 (0.4) | 4.7 (0.5) | 5.3 (0.3) | 4.8 (0.4) | 4.6 (0.4) | 4.5 (0.3) | 5.0 (0.3) | -0.02 | 0.735 |
|  |  | Cholesterol (mg/day) | 307.3 (53.6) | 423.9 (80.5) | 405.5 (62.1) | 350.8 (34.5) | 235.6 (32.3) | 272.8 (41.4) | 309.3 (36.0) | 355.4 (42.2) | -8.28 | 0.315 |
|  | **Normal** | Total sugars (%) | - | - | - | 14.4 (0.3) | 14.3 (0.3) | 13.7 (0.3) | 14.1 (0.3) | 13.7 (0.4) | -0.18 | 0.079 |
|  |  | Dietary fiber (g/day) | 17.3 (0.4) | 17.6 (0.5) | 17.1 (0.4) | 19.8 (0.4) | 19.8 (0.6) | 17.9 (0.5) | 18.1 (0.4) | 17.4 (0.4) | 0.09 | 0.146 |
|  |  | Saturated fatty acid (%) | 7.6 (0.1) | 7.9 (0.1) | 8.0 (0.1) | 8.3 (0.1) | 8.6 (0.2) | 9.0 (0.2) | 9.0 (0.2) | 8.7 (0.2) | 0.20 | <0.001 |
|  |  | MUFA (%) | 7.5 (0.1) | 7.9 (0.1) | 7.9 (0.1) | 7.3 (0.1) | 7.6 (0.2) | 8.1 (0.2) | 8.3 (0.1) | 8.4 (0.2) | 0.12 | <0.001 |
|  |  | PUFA (%) | 4.9 (0.1) | 5.1 (0.1) | 5.0 (0.1) | 5.2 (0.1) | 5.5 (0.1) | 5.4 (0.1) | 5.5 (0.1) | 5.8 (0.1) | 0.12 | <0.001 |
|  |  | N3 (%) | 0.6 (0.0) | 0.6 (0.0) | 0.6 (0.0) | 0.7 (0.0) | 0.7 (0.0) | 0.7 (0.0) | 0.7 (0.0) | 0.7 (0.0) | 0.02 | <0.001 |
|  |  | N6 (%) | 4.3 (0.1) | 4.5 (0.1) | 4.4 (0.1) | 4.5 (0.1) | 4.8 (0.1) | 4.7 (0.1) | 4.8 (0.1) | 5.1 (0.1) | 0.09 | <0.001 |
|  |  | Cholesterol (mg/day) | 285.6 (8.9) | 277.3 (8.6) | 295.7 (10.1) | 267.7 (8.0) | 268.7 (8.8) | 263.8 (12.8) | 278.9 (6.9) | 264.2 (7.4) | -2.82 | 0.035 |
| **Obesity**  **(weight)§** | **Obese** | Total sugars (%) | - | - | - | 13.9 (1.1) | 14.5 (1.2) | 12.1 (0.7) | 14.2 (1.2) | 12.6 (1.0) | -0.31 | 0.358 |
|  |  | Dietary fiber (g/day) | 18.4 (1.8) | 19.6 (2.1) | 17.1 (1.2) | 19.9 (1.5) | 19.4 (1.7) | 20.5 (3.0) | 21.2 (2.0) | 19.0 (1.4) | 0.25 | 0.366 |
|  |  | Saturated fatty acid (%) | 7.2 (0.5) | 8.5 (0.9) | 7.0 (0.5) | 8.5 (0.7) | 8.3 (0.5) | 8.3 (0.5) | 9.0 (0.6) | 9.4 (0.5) | 0.27 | 0.004 |
|  |  | MUFA (%) | 7.5 (0.5) | 7.6 (0.5) | 7.4 (0.6) | 7.9 (0.7) | 7.6 (0.6) | 7.6 (0.4) | 8.0 (0.6) | 9.5 (0.5) | 0.23 | 0.005 |
|  |  | PUFA (%) | 5.0 (0.3) | 5.0 (0.4) | 5.4 (0.5) | 5.9 (0.5) | 5.5 (0.3) | 5.0 (0.3) | 5.0 (0.4) | 5.9 (0.3) | 0.06 | 0.250 |
|  |  | N3 (%) | 0.7 (0.1) | 0.7 (0.1) | 0.6 (0.1) | 0.7 (0.1) | 0.8 (0.1) | 0.6 (0.1) | 0.7 (0.1) | 0.7 (0.1) | 0.01 | 0.493 |
|  |  | N6 (%) | 4.4 (0.3) | 4.3 (0.4) | 4.8 (0.4) | 5.2 (0.4) | 4.7 (0.3) | 4.4 (0.3) | 4.3 (0.3) | 5.2 (0.3) | 0.05 | 0.328 |
|  |  | Cholesterol (mg/day) | 297.7 (46.1) | 320.1 (45.3) | 353.9 (48.2) | 313.0 (38.2) | 265.9 (30.0) | 258.7 (34.3) | 284.4 (28.8) | 342.4 (38.5) | -1.52 | 0.810 |
|  | **Normal** | Total sugars (%) | - | - | - | 14.4 (0.3) | 14.3 (0.3) | 13.9 (0.3) | 14.0 (0.3) | 13.5 (0.4) | -0.21 | 0.041 |
|  |  | Dietary fiber (g/day) | 17.3 (0.4) | 17.6 (0.5) | 17.2 (0.4) | 20.0 (0.5) | 19.6 (0.6) | 18.1 (0.4) | 18.0 (0.4) | 17.4 (0.4) | 0.08 | 0.194 |
|  |  | Saturated fatty acid (%) | 7.6 (0.1) | 7.9 (0.1) | 8.0 (0.1) | 8.2 (0.1) | 8.6 (0.2) | 8.9 (0.2) | 8.9 (0.2) | 8.6 (0.2) | 0.18 | <0.001 |
|  |  | MUFA (%) | 7.5 (0.1) | 7.9 (0.1) | 7.9 (0.1) | 7.3 (0.1) | 7.6 (0.2) | 8.2 (0.2) | 8.3 (0.1) | 8.4 (0.2) | 0.11 | <0.001 |
|  |  | PUFA (%) | 4.9 (0.1) | 5.1 (0.1) | 5.0 (0.1) | 5.2 (0.1) | 5.5 (0.1) | 5.4 (0.1) | 5.5 (0.1) | 5.8 (0.1) | 0.11 | <0.001 |
|  |  | N3 (%) | 0.6 (0.0) | 0.6 (0.0) | 0.6 (0.0) | 0.7 (0.0) | 0.7 (0.0) | 0.7 (0.0) | 0.7 (0.0) | 0.7 (0.0) | 0.01 | <0.001 |
|  |  | N6 (%) | 4.4 (0.1) | 4.6 (0.1) | 4.4 (0.1) | 4.5 (0.1) | 4.8 (0.1) | 4.7 (0.1) | 4.8 (0.1) | 5.0 (0.1) | 0.09 | <0.001 |
|  |  | Cholesterol (mg/day) | 285.7 (9.0) | 280.7 (8.7) | 298.6 (10.3) | 269.8 (7.9) | 267.0 (8.8) | 264.1 (12.9) | 280.6 (7.2) | 264.4 (7.1) | -3.04 | 0.024 |
| **Obesity**  **(BMI)** **¶** | **Obese** | Total sugars (%) | - | - | - | 14.8 (1.0) | 13.8 (1.3) | 13.3 (1.0) | 12.4 (0.9) | 12.2 (0.8) | -0.65 | 0.031 |
|  |  | Dietary fiber (g/day) | 17.2 (1.6) | 19.7 (2.1) | 15.8 (1.0) | 19.8 (1.6) | 17.7 (1.7) | 19.1 (3.1) | 19.8 (1.9) | 18.4 (1.4) | 0.20 | 0.440 |
|  |  | Saturated fatty acid (%) | 7.3 (0.5) | 8.0 (0.7) | 6.9 (0.5) | 8.6 (0.7) | 8.4 (0.5) | 8.5 (0.6) | 8.4 (0.5) | 9.0 (0.5) | 0.23 | 0.006 |
|  |  | MUFA (%) | 7.4 (0.4) | 7.8 (0.6) | 7.3 (0.6) | 7.9 (0.8) | 7.6 (0.6) | 7.3 (0.4) | 8.0 (0.5) | 9.0 (0.5) | 0.18 | 0.024 |
|  |  | PUFA (%) | 5.3 (0.5) | 4.9 (0.4) | 5.0 (0.5) | 5.8 (0.5) | 5.4 (0.4) | 5.1 (0.3) | 5.2 (0.4) | 5.7 (0.4) | 0.05 | 0.445 |
|  |  | N3 (%) | 0.7 (0.1) | 0.6 (0.1) | 0.6 (0.1) | 0.7 (0.1) | 0.8 (0.1) | 0.6 (0.0) | 0.7 (0.2) | 0.7 (0.1) | 0.01 | 0.549 |
|  |  | N6 (%) | 4.7 (0.4) | 4.4 (0.4) | 4.5 (0.4) | 5.1 (0.4) | 4.5 (0.3) | 4.5 (0.3) | 4.5 (0.3) | 5.0 (0.4) | 0.03 | 0.566 |
|  |  | Cholesterol (mg/day) | 281.4 (44.7) | 334.4 (53.5) | 347.0 (50.6) | 277.1 (34.9) | 235.8 (29.0) | 245.5 (32.6) | 286.3 (35.3) | 316.8 (37.1) | -2.45 | 0.710 |
|  | **Normal** | Total sugars (%) | - | - | - | 14.3 (0.3) | 14.3 (0.3) | 13.8 (0.3) | 14.1 (0.3) | 13.5 (0.4) | -0.18 | 0.084 |
|  |  | Dietary fiber (g/day) | 17.3 (0.4) | 17.6 (0.5) | 17.2 (0.4) | 20.0 (0.5) | 19.7 (0.6) | 18.2 (0.4) | 18.1 (0.4) | 17.4 (0.4) | 0.09 | 0.146 |
|  |  | Saturated fatty acid (%) | 7.6 (0.1) | 8.0 (0.1) | 8.0 (0.1) | 8.2 (0.1) | 8.6 (0.2) | 8.9 (0.2) | 9.0 (0.2) | 8.7 (0.2) | 0.19 | <0.001 |
|  |  | MUFA (%) | 7.5 (0.1) | 7.9 (0.1) | 7.9 (0.1) | 7.3 (0.1) | 7.6 (0.2) | 8.2 (0.2) | 8.3 (0.1) | 8.5 (0.2) | 0.11 | <0.001 |
|  |  | PUFA (%) | 4.9 (0.1) | 5.1 (0.1) | 5.0 (0.1) | 5.2 (0.1) | 5.5 (0.1) | 5.4 (0.1) | 5.5 (0.1) | 5.8 (0.1) | 0.11 | <0.001 |
|  |  | N3 (%) | 0.6 (0.0) | 0.6 (0.0) | 0.6 (0.0) | 0.7 (0.0) | 0.7 (0.0) | 0.7 (0.0) | 0.7 (0.0) | 0.7 (0.0) | 0.02 | <0.001 |
|  |  | N6 (%) | 4.3 (0.1) | 4.6 (0.1) | 4.4 (0.1) | 4.6 (0.1) | 4.8 (0.1) | 4.7 (0.1) | 4.8 (0.1) | 5.1 (0.1) | 0.09 | <0.001 |
|  |  | Cholesterol (mg/day) | 286.4 (8.9) | 280.4 (8.6) | 299.1 (10.5) | 271.9 (8.0) | 268.6 (9.0) | 264.8 (12.9) | 280.7 (7.0) | 266.5 (7.5) | -2.89 | 0.032 |

SFA, saturated fatty acids; MUFA, monounsaturated fatty acids; PUFA, polyunsaturated fatty acids; N3, omega-3 fatty acids; N6, omega-6 fatty acids

*Survey-weighted linear regression was used to obtain beta coefficients and p-values.

†n = 11,816 for energy, carbohydrate, protein, and fat / n = 7,972 for fat subtypes and dietary fiber / n = 4,429 for total sugars

‡ n = 11,047 for energy, carbohydrate, protein, and fat / n = 7,435 for fat subtypes and dietary fiber / n = 4,738 for total sugars

§ n = 11,065 for energy, carbohydrate, protein, and fat / n = 7,446 for fat subtypes and dietary fiber / n = 4,438 for total sugars

¶ n = 11,063 for energy, carbohydrate, protein, and fat / n = 7,444 for fat subtypes and dietary fiber / n = 4,436 for total sugars
